# Mesoscopic sliding ferroelectricity enabled photovoltaic random access memory for material-level artificial vision system

**DOI:** 10.1038/s41467-022-33118-x

**Published:** 2022-09-14

**Authors:** Yan Sun, Shuting Xu, Zheqi Xu, Jiamin Tian, Mengmeng Bai, Zhiying Qi, Yue Niu, Hein Htet Aung, Xiaolu Xiong, Junfeng Han, Cuicui Lu, Jianbo Yin, Sheng Wang, Qing Chen, Reshef Tenne, Alla Zak, Yao Guo

**Affiliations:** 1 Beijing Institute of Technology, Haidian, Beijing, 100081 China; 2 Department of Electronics, Peking University, Haidian, Beijing, 100871 China; 3 Beijing Graphene Institute, Beijing, 100095 China; 4 Department of Molecular Chemistry and Materials Science, Weizmann Institute of Science, Rehovot, 760001 Israel; 5 Faculty of Sciences, Holon Institute of Technology, 52 Golomb St., Holon, 5810201 Israel

**Keywords:** Electronic devices, Electronic devices

## Abstract

Intelligent materials with adaptive response to external stimulation lay foundation to integrate functional systems at the material level. Here, with experimental observation and numerical simulation, we report a delicate nano-electro-mechanical-opto-system naturally embedded in individual multiwall tungsten disulfide nanotubes, which generates a distinct form of in-plane van der Waals sliding ferroelectricity from the unique combination of superlubricity and piezoelectricity. The sliding ferroelectricity enables programmable photovoltaic effect using the multiwall tungsten disulfide nanotube as photovoltaic random-access memory. A complete “four-in-one” artificial vision system that synchronously achieves full functions of detecting, processing, memorizing, and powering is integrated into the nanotube devices. Both labeled supervised learning and unlabeled reinforcement learning algorithms are executable in the artificial vision system to achieve self-driven image recognition. This work provides a distinct strategy to create ferroelectricity in van der Waals materials, and demonstrates how intelligent materials can push electronic system integration at the material level.

## Abstract (web summary)

Intelligent materials change their properties under external stimuli, integrating functionalities at the matter level. Here, Guo et al. report an artificial vision system based on the memory effect produced by sliding ferroelectricity in multiwalled tungsten disulfide nanotubes.

## Introduction

Since the demonstration of integrated circuits in the 1950s, the integration level of electronic systems has been remarkably developed and renovated^1^. On one hand, the success of Moore’s law enables the integration of billions of solid-state devices into a single die^2^, on the other, multiple functional submodules can be integrated into one chip or package, known as system on chip or system in package^3,4^. A typical functional system includes the sensing or input/output module for external information exchange, the processing module to execute the commands, the memory module to store the commands and data, and the power module to support the energy consumption^5^. Given the potential physical limit of Moore’s law, high-level system integration technology has drawn unprecedented attention. Non-von Neumann architecture, which is inspired by neuromorphic computing and collocates the memory and process modules, is promising to reduce data traffic, time delay, and energy cost^6,7^. Comparably, in-sensor computing integrates sensing and processing modules, and the sense and process can operate synchronously with an ultrafast speed in edge applications^8–10^. Further on, floating-gate devices based on two-dimensional materials with functions of sensing, memory, and processing enabled by photon-induced trap charge release were demonstrated, and the construction of an artificial retinomophic hardware system for motion detection was proposed^11,12^. With the explosive growth of data in the post Moore era, there is a great expectation for high-level system integration to further boost artificial intelligence and the internet-of-things. Herein a conceptual question is: which level can the system integration ultimately achieve?

From the perspective of material science, intelligent materials with controllable physical properties and reconfigurable responses to external stimuli are appropriate candidates to build functional electronic systems^13^. Ferroelectricity, which generates reversible spontaneous electric polarization with external voltage bias, is among the most preferred strategies to achieve memory in electronic devices^14–20^. Traditional ferroelectricity of both bulk and low-dimensional materials is generally regarded as the result of atomic position switching between the bi-stable states of the atomic unit cell structure^14–16^. The recently revealed parallelly stacked bilayer graphene or bilayer h-BN structures switching between AB and BA configuration was found to generate an interfacial out-of-plane spontaneous polarization^21–25^. Upon the centurial anniversary of ferroelectricity’s discovery^15–17^, it is of great significance to explore new forms of ferroelectricity, and fully play their memory function to meet the current challenges of electronics for the artificial intelligence era.

In this research, we report a mesoscopic in-plane sliding ferroelectricity that results from the superlubricity of the van der Waals interface^26–29^ and the piezoelectric nature of asymmetric structure of the WS_2_ nanotube^30–32^. This ferroelectricity is different from traditional ferroelectricity that originates from atomic lattice switching discovered 100 years ago, as well as the recently revealed interfacial ferroelectricity of parallelly stacked bilayer graphene or h-BN^14–17,21–25^. The mesoscopic sliding ferroelectricity produces programmable and nonvolatile photovoltaic effect in WS_2_ nanotubes, which are ideally suitable as photovoltaic random-access memory (PV-RAM). We further demonstrate a complete artificial vision system integrated in the ‘smart’ PV-RAM array, which enables synchronous multi-functions of sense, memory, compute, and power. Both labeled supervised learning and label-free reinforced learning are implantable in the PV-RAM system, which is self-driven and spontaneously outputs robust electrical signals with recognized image patterns.

## Results

**Fig. 1:**
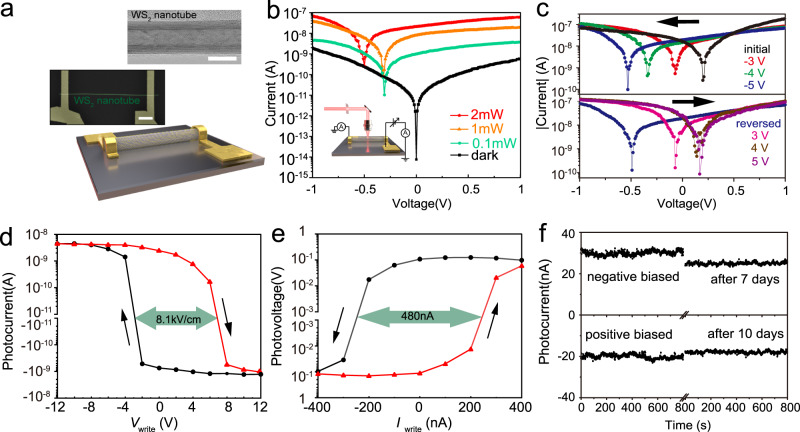
Programmability of the photovoltaic effect in WS_2_ nanotube. **a** Upper: transmission electron microscopy image of a multiwall WS_2_ nanotube, scale bar: 10 nm. Middle: scanning electron microscopy image (colored) of the WS_2_ nanotube device, scale bar: 2 μm. Lower: schematic of the WS_2_ nanotube device. **b** Log scale *I–V* curves with 633 nm laser spot focused at the middle of the channel with the power of 0 W, 0.1 mW, 1 mW, and 2 mW. Insert: Schematic diagram of the photoresponse measurement. **c** Programmable photovoltaic effect in WS_2_ nanotube with prior voltage bias of 0 V (initial), −3 V, −4 V, −5 V, −5 V (reversed), 3 V, 4 V, and 5 V, respectively, the black arrow indicates the test order. **d** Ferroelectric-like hysteresis loop of the photovoltaic effect through voltage sweep. **e** Ferroelectric-like hysteresis loop of the photovoltaic effect through current sweep. **f** Nonvolatility of the programmed photocurrent.

High-quality multiwall WS_2_ nanotubes were synthesized and the devices were fabricated, as shown in Fig. 1a and Supplementary Fig. 1. The spontaneous photovoltaic effect of WS_2_ nanotubes has been revealed in a previous report^33^, which is well reproduced in Fig. 1b and Supplementary Fig. 2. The photo absorption and photoelectrical response of WS_2_ nanotube cover the nearly full red to blue visible band, which makes WS_2_ nanotube suitable candidate to build the bottom devices for artificial vision systems, as shown in Supplementary Fig. 3 to Supplementary Fig. 8. The linearity of the photoresponse is evaluated in Supplementary Figs. 9 and 10. More interestingly, we discover that the photovoltaic effect of the WS_2_ nanotubes is programmable. As shown in the upper Fig. 1c, initially, the photovoltage of a WS_2_ nanotube device is 0.21 V and the photocurrent is −15.7 nA. By applying various negative voltages bias of −3 V, −4 V, and −5 V for 5 s prior to the *I–V* tests, the *I–V* curves shift and the photovoltage steps down to −0.07 V, −0.34 V, and −0.53 V, and the photocurrent increases to 6.0 nA, 14.3 nA, and 19.1 nA, respectively. The direction of the photovoltaic effect is reversed. Further on, the photovoltage and photocurrent can be reversed back by applying positive voltage. As shown in the lower Fig. 1c, by applying voltage bias of 3 V, 4 V, and 5 V to the same device, the photovoltage step from −0.49 V to −0.07 V, 0.14 V, and 0.17 V, and the photocurrent decrease from 18.6 nA to 5.3 nA, −23.9 nA, and −26.0 nA, respectively. The linear plot of the *I–V* curves is shown in Supplementary Fig. 11. Therefore, the photovoltaic effect of the WS_2_ nanotube is adjustable with a prior bias, rather than having a fixed direction and magnitude. The reversion of the photovoltaic effect is reproducible and is obtained on multiple WS_2_ nanotube devices, as shown in Supplementary Fig. 12. We also observe that there are devices with indistinctive initial photovoltaic effect, still, the application of prior bias can generate the programmable photovoltaic effect. Besides the photovoltaic effect, the prior bias also alters the rectification behavior of the WS_2_ nanotube devices as shown in Supplementary Fig. 13, which indicates the change of the electrostatic status inside the nanotube, as we will discuss later in this article.

To further exhibit the programmability of the photovoltaic effect, we apply equidistant looping bias (as writing voltage *V*_write_) to the WS_2_ nanotube, and collect the corresponding photocurrent *I*_ph_ with zero bias (*V*_write_ retracted). As shown in Fig. 1d, the *I*_ph_–*V*_write_ curve shows a ferroelectric-like hysteresis. The extracted coercivity is 9.8 V, or 8.1 kV/cm. Such a coercive electric field is relatively low compared to typical ferroelectric materials, which, for example, is around 40 kV/cm for BiFeO_3_ and Pb(Zr_0.4_Ti_0.6_)O_3_^34–36^. Similarly, the photovoltaic response can be also programmed with the current bias, as shown in Fig. 1e, the *V*_ph_–*I*_write_ curve has a coercivity of 480 nA. The programmed photovoltaic effect is non-volatile and can be maintained for at least a few days, as shown in Fig. 1f.

**Fig. 2:**
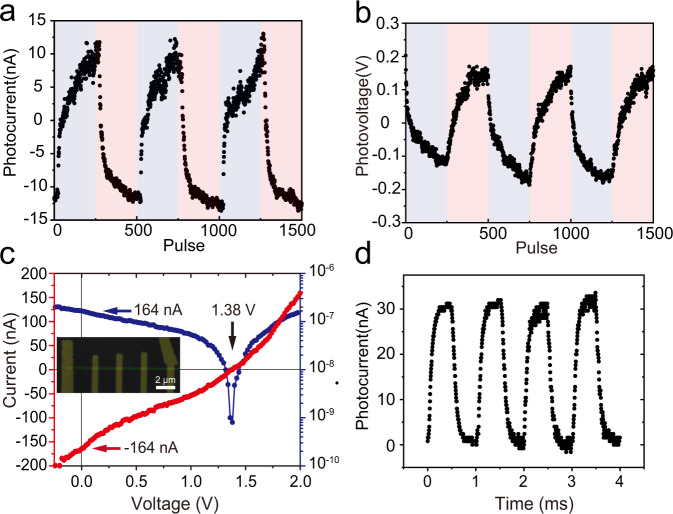
Continuous programmability and additivity of the photovoltaic effect. **a** Photocurrent programmed by voltage pulses. **b** Photovoltage programmed by current pulses. **c** *I–V* curve of the WS_2_ nanotube devices consisting of 4 series channels. **d** The photoresponse of the WS_2_ nanotube with 532 nm laser.

We further show the programmability of the photovoltaic effect of WS_2_ nanotubes through the pulsed mode. As shown in Fig. 2a, by applying stimulating voltage pulses (±6 V, 5 μs) to the WS_2_ nanotube, the subsequent short circuit *I*_ph_ can be reversed and continuously modulated. The negative pulses increase *I*_ph_, and the positive pulses decrease *I*_ph_, and the direction of the *I*_ph_ can be reversed. The continuous programmability of the photovoltaic effect enables multilevel memories. Similarly, the photovoltaic effect can also be programmed with current pulses (±500 nA, 5 μs), as shown in Fig. 2b. The energy consumption to reverse the direction of the photovoltaic effect is estimated at a few hundred nJ (voltage × current × pulse width × pulse number). The programmable photovoltaic response achieved by the compact two-terminal WS_2_ nanotube device is comparable to resistive random access memory (RRAM), magnetic random access memory (MRAM), or phase change random access memory (Pc-RAM)^37–40^ These three types of memories use different resistive, magnetic, or phase states to achieve non-volatile memory. Here, we demonstrate the programmability of the photovoltaic effect of the WS_2_ nanotube device and describe it as a photovoltaic random access memory (PV-RAM). Compared to the fixed but unpredictable photovoltaic effect, the determinatively programmable photovoltaic effect with controlled sign or direction, allow photocurrent or photovoltage accumulation using WS_2_ nanotubes connected in parallel or in series. This demonstration is an important advance toward its practical application whether for energy harvesting or edge computing, as is shown in Supplementary Fig. 14. Given the intact length of the WS_2_ nanotube, the photovoltaic effect can also be multiplicated by adding virtual electrodes to the channel^41^. As shown in Fig. 2c, the *V*_ph_ of the device with four series channels reach up to 1.38 V with a 10 mW laser illumination on the whole device area. The photoresponse of the WS_2_ nanotube with a chopped 532 nm laser is shown in Fig. 2d and Supplementary Fig. 15, from which we extracted the rise time of 64 μs and fall time of 77 μs. We list the photovoltaic performances of the WS_2_ nanotube device and a few low-dimensional material devices in previous studies, as is shown in Supplementary Table 1.

**Fig. 3:**
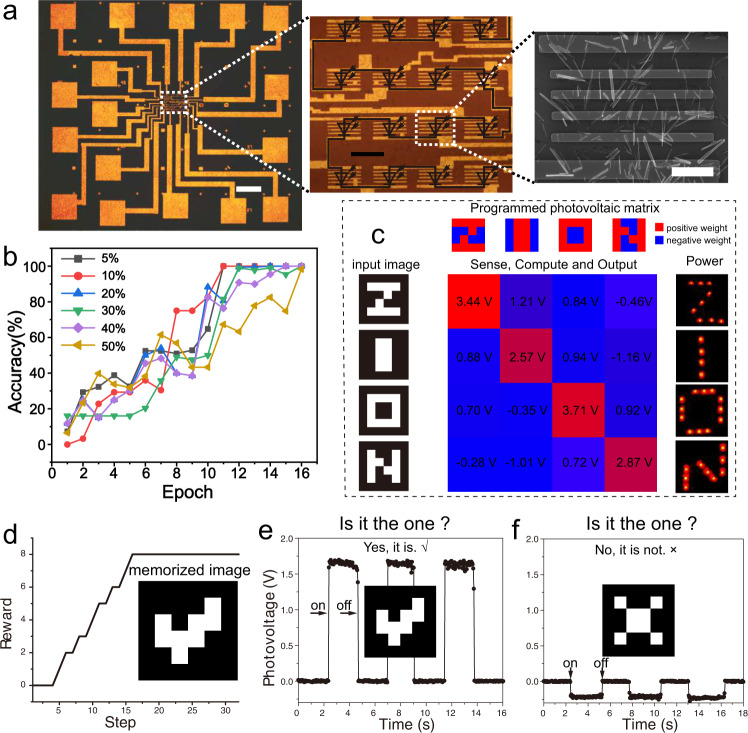
Full-functional artificial vision system implanted in PV-RAM array. **a** Optical microscopy images (left and middle) and scanning electron microscopy image of the PV-RAM array. Scale bar, left: 100 μm, middle: 20 μm, right: 5 μm. **b** Learning curve of accuracy with different levels of noise. **c** Demonstration of the artificial vision system. The binary weights were presented by the programmed photoresponse. The input images of “Z”, “I”, “O”, “N” were projected onto the array by a 200 mW laser. The output voltages were collected, which indicates the probability. The output can flicker LED patterns through a capacitor. **d** The off-line reinforcement learning process. **e**, **f** Recognition of the learned image (√, **e**) from other image (×, **f**).

The PV-RAM, which has a programmable and non-volatile photovoltaic effect, lays the foundation for building a complete artificial vision system that achieves multi-functions of sense, memory, computing, and power. A 4 by 4 PV-RAM pixel array was fabricated on randomly dispersed WS_2_ nanotubes, as shown in Fig. 3a and Supplementary Fig. 16. Each pixel has a size of 20 × 20 μm^2^ and contains multiple WS_2_ nanotube channels. The PV-RAMs were connected in series to sum the photovoltages, and the output voltage *V*_ph_ = Σ(**R**_4×4_*P_4×4_) performs a multiplication and sum operation (a convolution integral), with **R**_4×4_ being the photovoltage responsivity matrix that represents the weights, **P**_4×4_ being the photo intensity on each pixel as input. We note that there have been methods that massively fabricate carbon nanotube network devices^42–44^. Homogeneous WS_2_ nanotube networks can be obtained with further investigation into the synthesis, purification, dispersion, and fabrication processes. Here we focus on the photoelectrical behavior of the WS_2_ nanotube PV-RAM and prototype the integration of the artificial system into the PV-RAMs.

Firstly, we aim to achieve image identification of the letters ‘ZION’ with labeled training. We use off-line training with discrete binary and continuous weight, as shown in Fig. 3b, Supplementary Figs. 17 and 18. The trained weights are shown in Supplementary Figs. 19, 20. The PV-RAMs were then programmed according to the trained weight, and the image patterns were projected onto the array by a 532 nm laser through a customized pre-pattern mask. As shown in Fig. 3c, the WS_2_ nanotube PV-RAM array, programmed following the binary weights, outputs a voltage that indicates the probability of the recognized pattern through a SoftMax function (see Supplementary Fig. 21). The magnitude of the output voltage of up to >2.5 V can further charge a capacitor (1 μF) and flicker LEDs fixed on office paper as flexible substrate (see Fig. 3c right, Supplementary Figs. 22 and 23). Therefore, the PV-RAM array can maintain the pre-set weight (memory), detect the light (sense), recognize the image pattern (compute), and spontaneously output a robust voltage that can drive secondary-level electronic devices (power). It is conceptually straightforward to further scale the pixels to larger dimensions or to multi-color, for example, Red-Green-Blue channels, as a future technological task.

Besides the labeled supervised learning, weights from unlabeled reinforced learning are also implantable in the PV-RAM array^45^. The training policy of the reinforced learning is to adjust the weight in the neural network (photoresponse of each PV-RAM) to maximize the reward function, which is set *Re* = *V*_output_^pattern^−*V*_output_^all^/2 in this case. Here *V*_output_^pattern^ is the output voltage with the laser pattern projected on the array (a ‘√’), and *V*_output_^all^ is the output voltage with all PV-RAM in the array illustrated. Such a training strategy actually makes the PV-RAM array ‘remember’ the projected image. Figure 3d shows the learning curve of the reinforced learning. By programming the learned binary weight, the PV-RAM array can well recognize the previously memorized pattern (the ‘√’) from random patterns (for example, a ‘×’), as shown in Fig. 3e, f, respectively.

Lastly, we discuss the mechanism for the programmability of the photovoltaic effect in WS_2_ nanotube. In the previous study, asymmetric van der Waals layers were shown to induce shift current and generate photovoltaic effect with fixed direction^33,46^. The shift current is an important physical mechanism to generate the bulk photovoltaic effect with a fixed direction^47^. The photovoltaic effect in this work, which is programmable in both direction and magnitude by bias, should be dominated by a different mechanism. Moreover, the shift current induced anomalous photovoltaic effect would generate an open circuit photovoltage that is linearly dependent of the channel length^48^. However, as shown in Supplementary Fig. 24, the experimental results show that the *V*_ph_ is obviously independent of the channel length. Therefore, the shift current might contribute to the photocurrent in the WS_2_ nanotube to a certain degree, the overall photovoltaic effect, which switches its direction by the external voltage bias, should be dominated by the ferroelectric-like mechanism that switches the photovoltaic effect of WS_2_ nanotube.

**Fig. 4:**
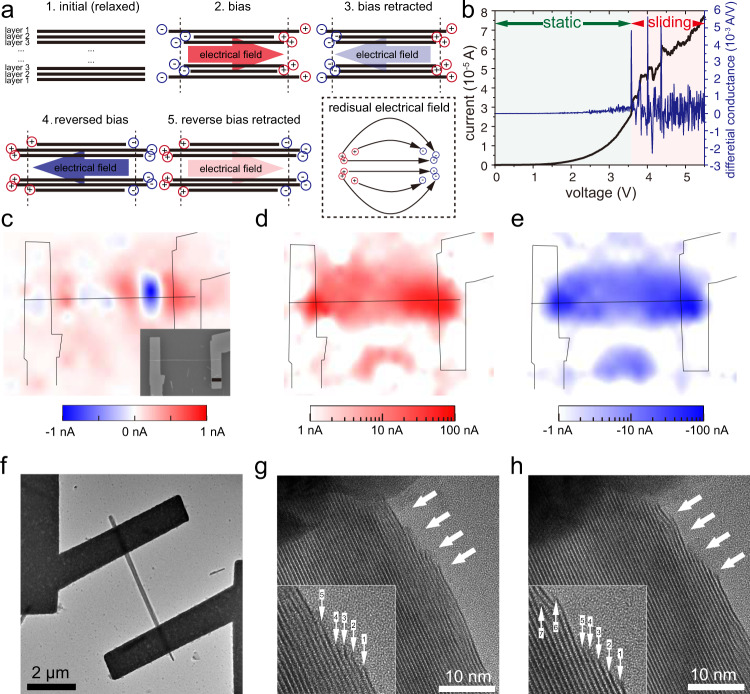
Mechanism of the programmable photovoltaic effect. **a** Sliding between the WS_2_ layers, which generates residual strain and a spontaneous electric field. **b** Current and differential conductance versus voltage bias. **c** Mapped photocurrent of a WS_2_ nanotube device before bias. The scale bar is 2 μm. **d**, **e** Mapped photocurrent of the WS_2_ nanotube device after negative bias (**d**) and after positive bias (**e**). Note the different scales of **c**–**e**. The laser intensity is 1 mW. **f** A WS_2_ nanotube device. **g**, **h** The layered structure of the WS_2_ nanotube before (**g**) and after (**h**) bias.

Given the superlubricity between the incommensurate van der Waals interface and the piezoelectric nature of monolayer WS_2_ sheets^23–25,30–32^, we propose a mesoscopic van der Waals sliding ferroelectric effect for the WS_2_ nanotube. As shown in Fig. 4a, Supplementary Fig. 25 and Supplementary Movies 1 and 2, the WS_2_ nanotube consists of multiple cylindrical incommensurate layers that lack inversion symmetry, each layer has random piezoelectricity along the axial direction, depending on their chirality^30,31^. By applying a voltage bias, the layers of WS_2_ with random piezoelectricity tend to extend or shrink along the axis due to the reverse piezoelectric effect^32^. The neighboring layers with different piezoelectricity might tend to deform in different magnitude or in opposite directions (for example, layers with positive piezoelectric coefficient tend to extend, while layers with negative piezoelectric coefficient tend to shrink). With a larger voltage bias, the shear force can exceed the low friction force between the layers and the layers will slide over to each other to extend or shrink, as shown in step 2 of Fig. 4a^26–28,49–51^. After the withdrawal of the bias, the layers will tend to recover from the deformation, but the friction force will prevent the full recovery, leaving the residual deformation and strain in the WS_2_ nanotube. Due to the direct piezoelectric effect, the residual deformation will generate spontaneous electrical polarization. What is important here is that the deformed layers, whether shrunken or extended due to the opposite reverse piezoelectric coefficient, will generate electrical polarization in the same direction, and therefore generate net residual electrical polarization due to the opposite direct piezoelectricity coefficient (see step 3). A similar analysis applies to the reverse bias (see steps 4 and 5). As such, each individual WS_2_ nanotube is functionalized as a dedicated “nano-electro-mechanical-system”, or NEMS, plus the photovoltaic capability. The residual strain-induced piezoelectric charge concentrates at the opposite ends of the nanotube, which is similar to separated opposite point charges and generates non-volatile polarization, i.e., the van der Waals sliding ferroelectricity or sliding ferroelectricity. We note that the appropriate friction between the curved van der Waals layers is important for the preservable sliding ^29,52^, as is shown in Supplementary Fig. 26. If there was no friction force, the sliding would not be preserved after the withdrawal of the bias, while if the friction force was too large, the interlayers are locked and sliding would hardly take place. The sliding ferroelectricity is in accordance with the experimental observations that current fluctuations dramatically increase beyond a threshold voltage, indicating the sliding of the layers inside the WS_2_ nanotube (Fig. 4b and Supplementary Fig. 27). We also mapped the photocurrent of the WS_2_ device before (Fig. 4c) and after negative/positive bias (Fig. 4d, e). The channel length is >10 μm, which provides adequate spatial resolution with the laser spot size of around 1 μm. Initially, the photocurrent is small and has varying directions along the WS_2_ nanotube. After the positive/negative bias, we observe negative/positive photocurrent to be more intense at the end of the device, rather than at the middle of channel, which is in accordance with the simulated residual charge and voltage potential distribution, as shown in Supplementary Fig. 25 (also see discussion about the concerned Schottky contact in Supplementary Text)^53^. The reversion of the photocurrent is in accordance with the Kelvin probe force microscopy (KPFM) characterized potential distribution of the WS_2_ nanotube device channel, as shown in Supplementary Fig. 28. To further confirm the interlayer sliding of the WS_2_ nanotube, we conducted an in situ TEM characterization. We fabricated the WS_2_ nanotube device on a suspending SiN_x_ film and transferred it into the TEM chamber for further characterization, as shown in Supplementary Fig. 29. By applying the positive/negative voltage of 10 V to the WS_2_ nanotube device, the occurrence of the sliding is observed, as shown in Fig. 4f–h. As marked by the arrows, terminal edges of layers, used as visual marks in the TEM characterization, change their relative positions and provide a direct evidence for the bias induced interlayer sliding of the WS_2_ nanotube layers.

The electric-driven sliding between van der Waals layers was also recently reported in parallelly stacked bilayer h-BN^23^, which generates out-of-plane ferroelectricity. However, please note that these are two fundamentally different kinds of ferroelectricity. The h-BN switches from AB to BA lattice configuration through atomic-scale sliding only in the parallel stacks and generates an interfacial out-of-plane polarization^22–25^. Here the cylindrical incommensurate WS_2_ layers slide by a mesoscale norm, generate an axis parallel in-plane polarization through the reverse piezoelectric effect, and further give rise to the programmable photovoltaic effect. The immigration of defects such as intercalated oxygen ion or vacancy might also contribute to an electrostatic redistribution, however, the relatively low mobility of ions or vacancies migration is inconsistent with the observed fast current changes (see Supplementary Fig. 27) and photovoltaic effect reversion in tens of μs (Fig. 2a, b)^54^.

## Discussion

To summarize, this work reports the mesoscopic scale sliding ferroelectricity that enables a naturally embedded nano-electro-mechanical-opto-system with programmable photovoltaic effect in multiwall van der Waals WS_2_ nanotube, and prototypes the full functional artificial vision system integrated into the WS_2_ nanotube network. The sliding ferroelectricity, as a consequence of the mesoscopic scale superlubricity and the piezoelectricity (sliding ferroelectricity = superlubricity + piezoelectricity), is a form of spontaneous polarization that differs from the traditional ferroelectricity that arises from atomic scale ionic position shift. The mesoscopic scale sliding ferroelectricity could be further extended to other incommensurate van der Waals layers. The artificial vision system, integrated down to the material level, achieves full functions of sensing, computing, memorizing, and powering. This work provides a distinct strategy to generate ferroelectricity and programmable photovoltaic effect, and prototypes of how intelligent materials can push the integration of electronic systems towards an ultimate level.

## Methods

### Materials

Multiwall WS_2_ nanotubes were synthesized by a two-step reaction route, when initially grown tungsten suboxide nanowhiskers are, subsequently, sulfurized into WS_2_ nanotubes. Both reactions took place in the same reactor and under the same conditions and follow each other via a self-controlled process (a one-pot reaction). In particular, oxide nanoparticles, the precursor for this reaction, consisted of a mixture of different WO_*x*_ (2.83 ≤ *x* ≤ 3) suboxide phases and morphologies. The nanowhiskers grow via a sequence of intermediate reactions including reduction of the precursor into volatile suboxide phase (WO_2.75_) and its evaporation, an additional partial reduction of the vapor into WO_2_ and condensation of the vapor mixture into 1D nanocrystals through formation of a stable W_18_O_49_ suboxide phase. These suboxide nanowhiskers are converted into WS_2_ nanotubes starting from the surface continuing inwards towards the inner core via slow diffusion-controlled sulfurization reaction. The majority of the nanotubes range from 2 to 20 μm in length and from 20 to 150 nm in diameter (see Supplementary Fig. 1) with a mean diameter of 70 nm.

### Devices and characterizations

WS_2_ nanotubes were dispersed onto Si/SiO_2_ (500 nm) wafer, and their positions were identified through optical microscopy. Electrodes were defined by electron beam lithography and electron beam evaporation of 20 nm Cr/120 nm Au or 20 nm Ti/120 nm Au followed by a lift-off process in acetone. Optical-electrical characterizations were performed with a combination of Bruker and Horiba Raman microscopes, Keithley semiconductor characterization systems, source meters, current-voltage amplifier, and oscilloscope. The 785, 633, 532, 488, 325 nm, and continuous wave lasers were implanted in the Raman microscope. The laser power was set 1 mW if not specified. For the PV-RAM array characterization, a commercial 532 nm diode-pumped solid-state laser of up to 200 mW was used to project image patterns through a customized mask, each PV-RAM was wire-bonded through a circuit control box to connect the external writing voltage, and the overall photovoltage was obtained from the outmost electrodes of the PV-RAM series. The in situ TEM sample was prepared as described in the supplementary information, and the characterization was performed with an FEI Tecnai G2 F20. The KPFM characterization was performed with a Bruker multi mode 8 AFM.

## Supplementary information

Supplementary Information

Description of Additional Supplementary Files

Supplementary Movie 1

Supplementary Movie 2

## Data Availability

The data used in this study are available in the Zenodo database^55^ with open access [10.5281/zenodo.6970428].

## Code availability

The code that supports the findings of this study is available in Github https://github.com/codestonano/Zion.
