# Supplementary material for: Mesoscopic sliding ferroelectricity enabled photovoltaic random access memory for material-level artificial vision system
Source: Nat Commun. 2022 Sep 14;13:5391. doi: 10.1038/s41467-022-33118-x (PMC9474805; doi:10.1038/s41467-022-33118-x)
Supplement: Supplementary file 1 — Supplementary Information [file 41467_2022_33118_MOESM1_ESM.pdf]

Supplementary Information for

Mesoscopic sliding ferroelectricity enabled photovoltaic random access memory for material-level artificial vision system

Yan Sun[1], Shuting Xu[1], Zheqi Xu[1], Jiamin Tian[2], Mengmeng Bai[1], Zhiying Qi[1], Yue Niu[1], Hein Htet Aung[1], Xiaolu Xiong[1], Junfeng Han[1], Cuicui Lu[1], Jianbo Yin[3], Sheng Wang[2], Qing Chen[2], Reshef Tenne[4], Alla Zak[5*], Yao Guo[1*]

[1]Beijing Institute of Technology; Haidian, Beijing, 100081, China.

[2]Department of Electronics, Peking University; Haidian, Beijing, 100871, China.

[3]Beijing Graphene Institute, Beijing, 100095, China.

[4]Department of Molecular Chemistry and Materials Science, Weizmann Institute of Science, Rehovot 760001, Israel.

[5]Faculty of Sciences, Holon Institute of Technology, 52 Golomb St., Holon 5810201, Israel.

Email: alzak@hit.ac.il; yaoguo@bit.edu.cn

These authors contributed equally: Y.S., S.X, Z.X.

Table of content

Supplementary Fig. 1. WS_2 nanotubes synthesized by two-step reaction route ... 3

Supplementary Fig. 2. Photovoltaic effect of WS_2 nanotube ... 4

Supplementary Fig. 3. Photocurrent spectrum of the pristine WS_2 nanotubes ... 5

Supplementary Fig. 4. Photovoltaic measurements with 785 nm laser ... 6

Supplementary Fig. 5. Photovoltaic measurements with 633 nm laser ... 7

Supplementary Fig. 6. Photovoltaic measurements with 532 nm laser ... 8

Supplementary Fig. 7. Photovoltaic measurements with 488 nm laser ... 9

Supplementary Fig. 8. Photovoltaic measurements with 325 nm laser ... 10

Supplementary Fig. 9. Dependence of photocurrent on different laser power ... 11

Supplementary Fig. 10. Photocurrent and photovoltage with different laser power ... 12

Supplementary Fig. 11. I-V curves in linear scale ... 13

Supplementary Fig. 12. Switchable photovoltaic effect in 9 WS_2 nanotube devices ... 14

Supplementary Fig. 13. I-V curves in dark after applying positive and negative bias, which show switchable rectification ... 15

Supplementary Fig. 14. Overall photocurrent of parallel photovoltaic devices with determinative/random direction ... 16

Supplementary Fig. 15. Photo response to a single laser ON/OFF period ... 17

Supplementary Table. 1 Photovoltaics of 2D MoS_2, 2D WS_2, 2D WSe_2, 1D WS_2, and BiFeO3. Only photovoltaic performance is included. ... 18

Supplementary Fig. 16. Fabrication process of the WS_2 nanotube PV-RAM array ... 19

Supplementary Fig. 17. Learning curve of loss with different levels of noise ... 20

Supplementary Fig. 18. The training process of the neural network with continuous weight. Red: accuracy. Blue: Loss function. Noise level is set 0 ... 21

Supplementary Fig. 19. The trained weights of the artificial neural network ... 22

Supplementary Fig. 20. The trained weights of the binary artificial neural network ... 23

Supplementary Fig. 21. Classification of the images ... 24

Supplementary Fig. 22. Equivalent circuits of the PV-RAM array, which can charge the capacitor and drive LED patterns ... 25

Supplementary Fig. 23. LED patterns of ‘Z’, ‘I’, ‘O’, and ‘N’ fixed by electrargol on office paper as the flexible substrate ... 26

Supplementary Fig. 24. Photovoltaic effect of WS_2 nanotube devices with different channel lengths ... 27

Supplementary Fig. 25. The simulated sliding process between WS_2 layers that generates ferroelectricity ... 28

Supplementary Fig. 26. Model calculations of interlayer sliding under different friction conditions ... 29

Supplementary Fig. 27. I-V curve and differential conductance of the WS_2 nanotube ... 30

Supplementary Fig. 28. Spontaneous electrical polarization of WS_2 nanotubes under KPFM ... 31

Supplementary Fig. 29. Fabrication process of the WS_2 nanotube for the in-situ TEM characterization ... 32

Supplementary Text ... 33

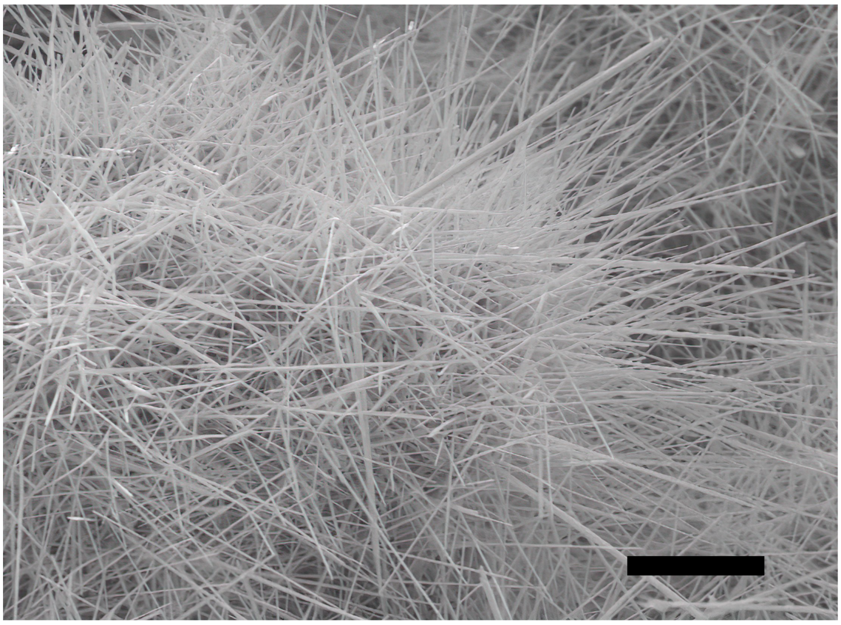

Supplementary Fig. 1 | WS_2 nanotubes synthesized by two-step reaction route. The majority of the nanotubes range from 2 to 20 μm in length and from 20 to 150 nm in diameter with the mean value of 70 nm. The scale bar is 5 μm.

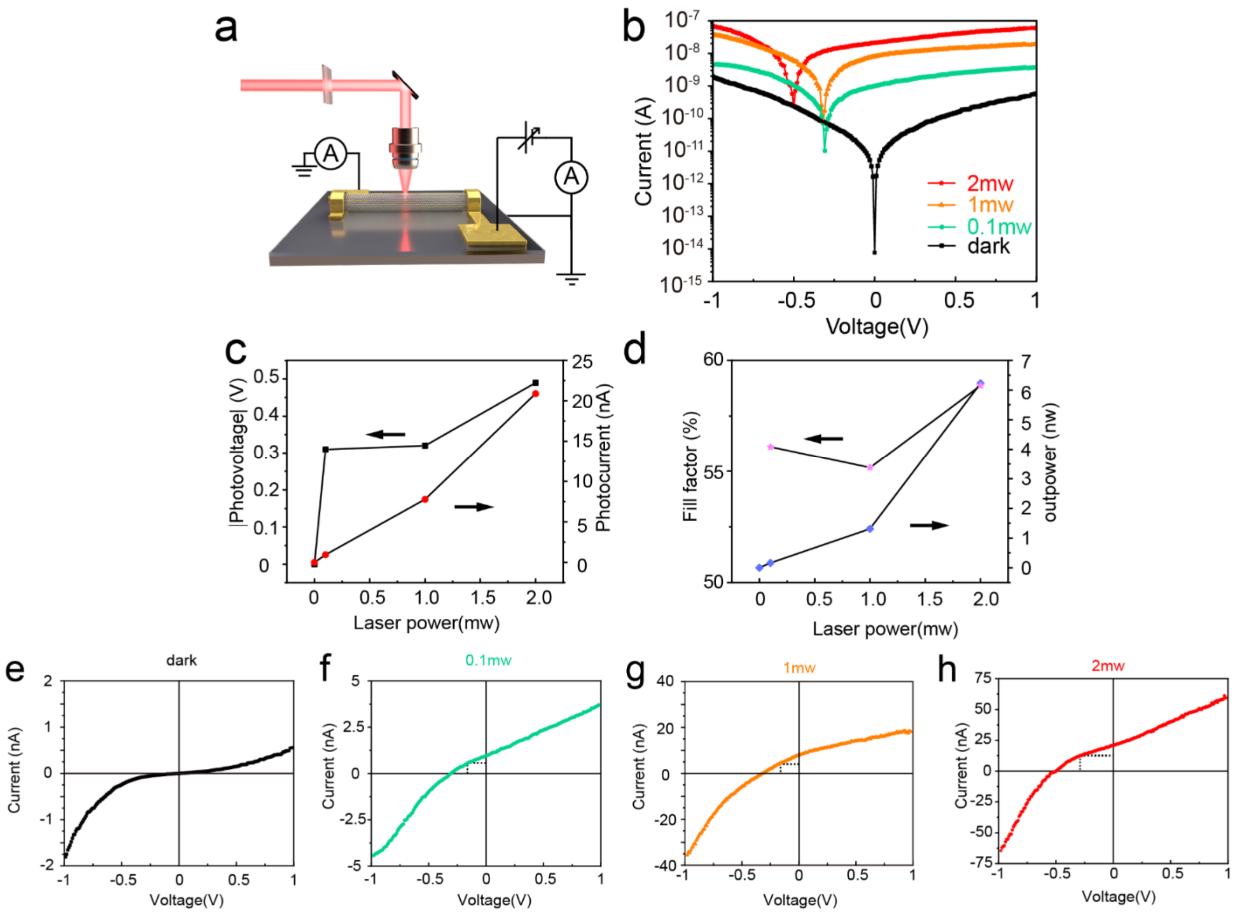

Supplementary Fig. 2 | Photovoltaic effect of WS_2 nanotube. a, Schematic diagram of the photoresponse measurement. **b**, Log scale *I-V* curves with 633 nm laser spot focused at the middle of the channel with the power of 0 W, 0.1 mW, 1 mW, and 2 mW. **c**, Photovoltage and photocurrent extracted from the *I-V* curves. **d**, Fill factor and output power extracted from the *I-V* curves. **e-h**, Linear scale *I-V* curves.

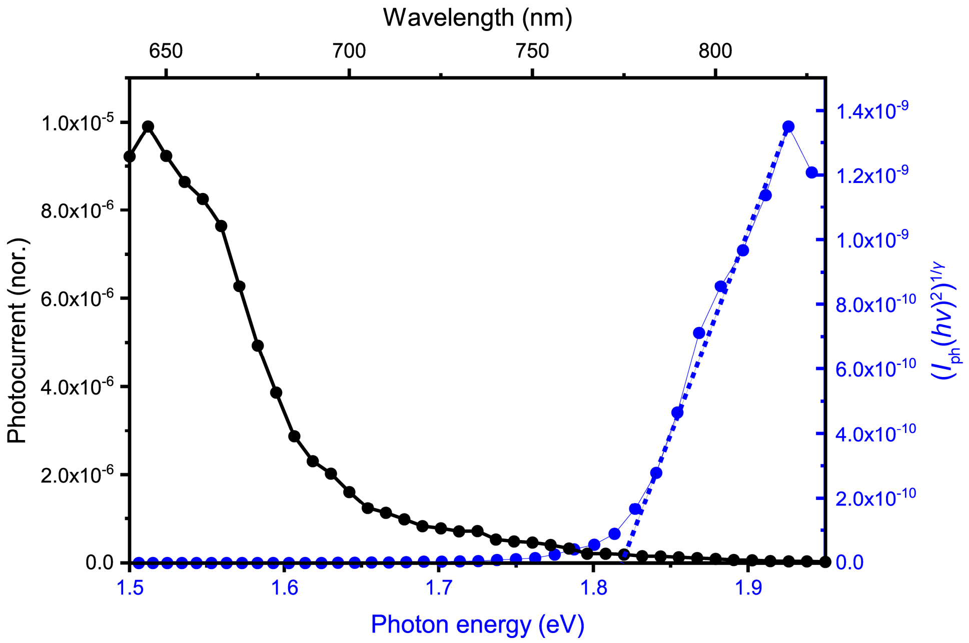

Supplementary Fig. 3 | Photocurrent spectrum of the pristine WS_2 nanotubes. The photocurrent spectroscopy study from 640 nm to 830 nm covers the absorption edge of the WS_2 nanotube. In accordance with the absorption spectroscopy, the photocurrent spectroscopy shows that the bandgap is about 1.8 eV.

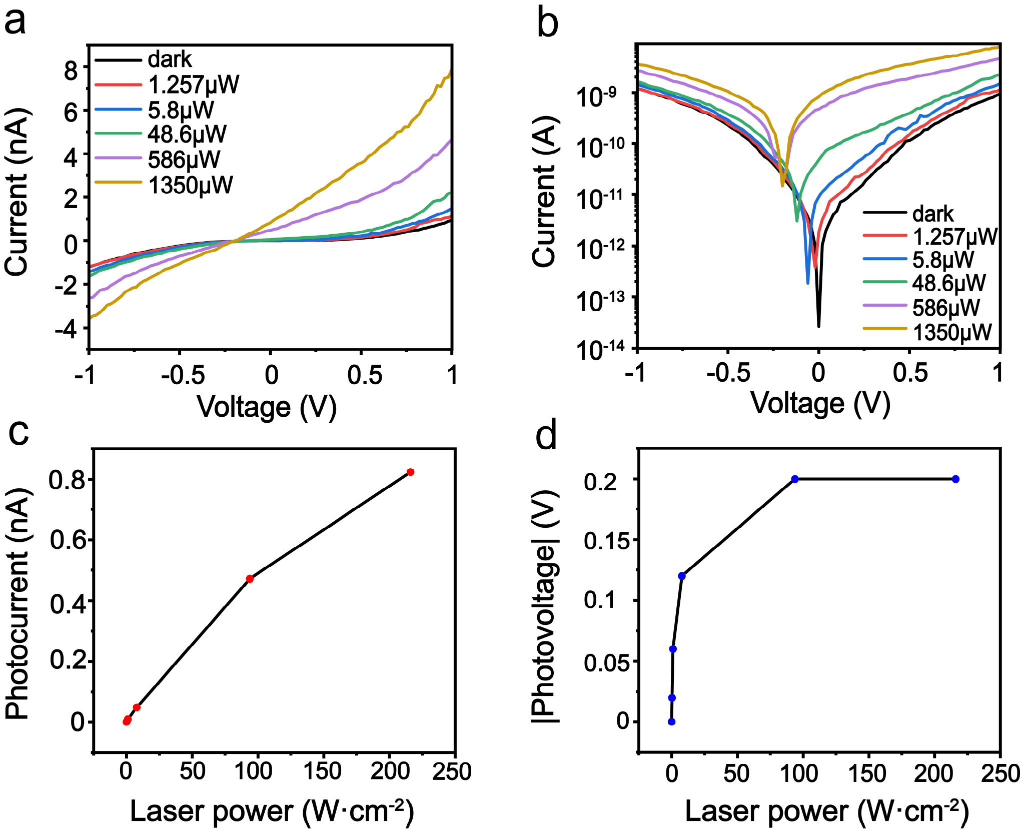

Supplementary Fig. 4 | Photovoltaic measurements with 785 nm laser. a, b, Linear (a) and log (b) scale *I-V* curves of the WS_2 nanotube. **c, d**, Photocurrent (c) and photovoltage (d) extracted from the *I-V* curves.

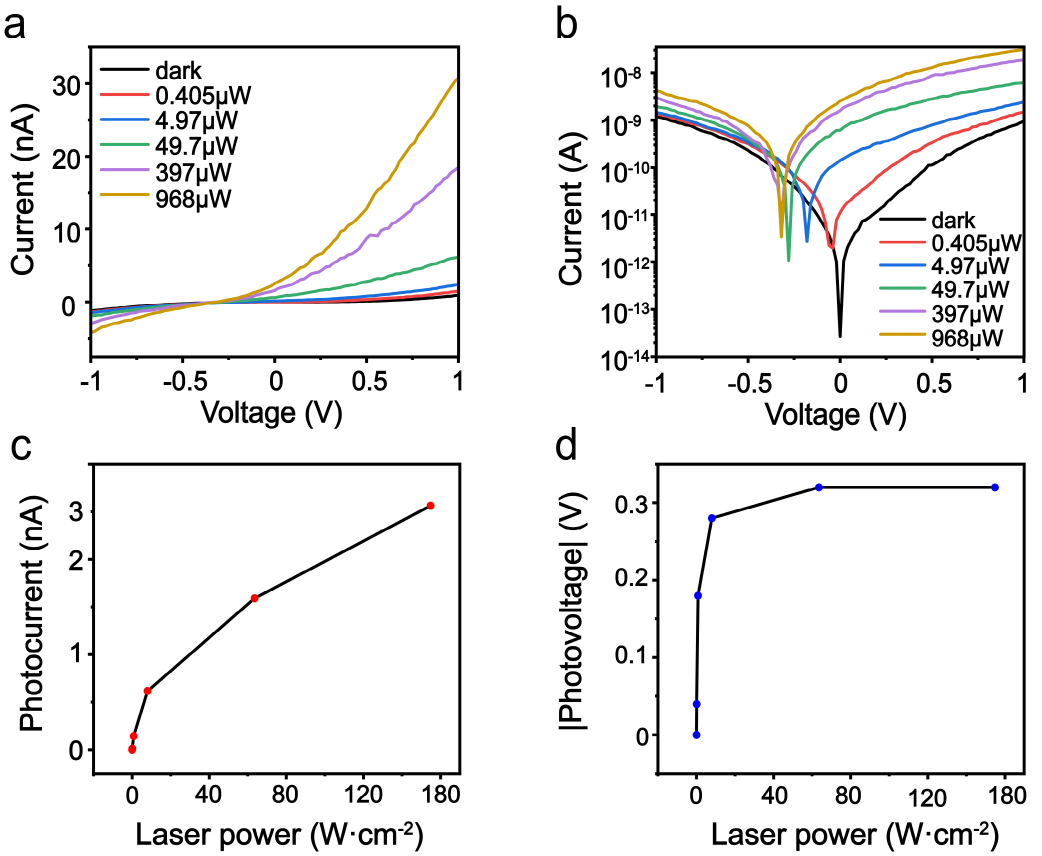

Supplementary Fig. 5 | Photovoltaic measurements with 633 nm laser. a, b, Linear (a) and log (b) scale *I-V* curves of the WS_2 nanotube. **c, d**, Photocurrent (c) and photovoltage (d) extracted from the *I-V* curves.

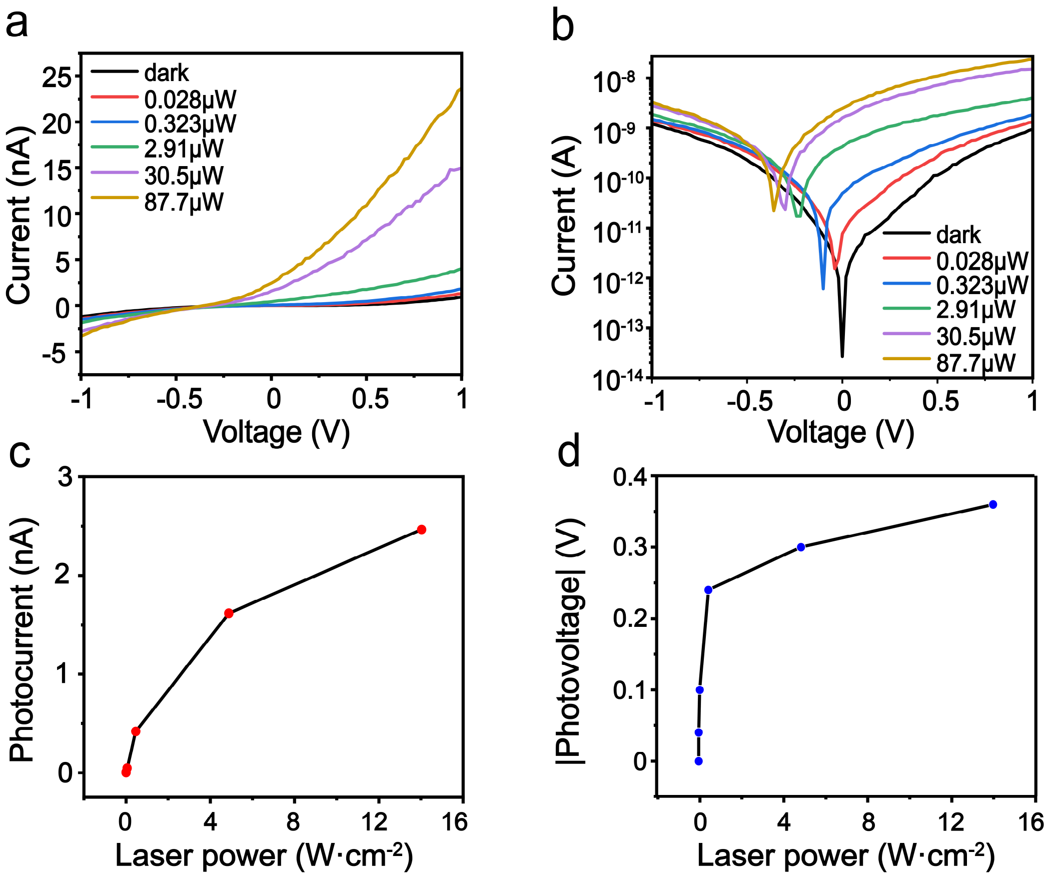

Supplementary Fig. 6 | Photovoltaic measurements with 532 nm laser. a, b, Linear (a) and log (b) scale *I*-*V* curves of the WS_2 nanotube. **c, d**, Photocurrent (c) and photovoltage (d) extracted from the *I*-*V* curves.

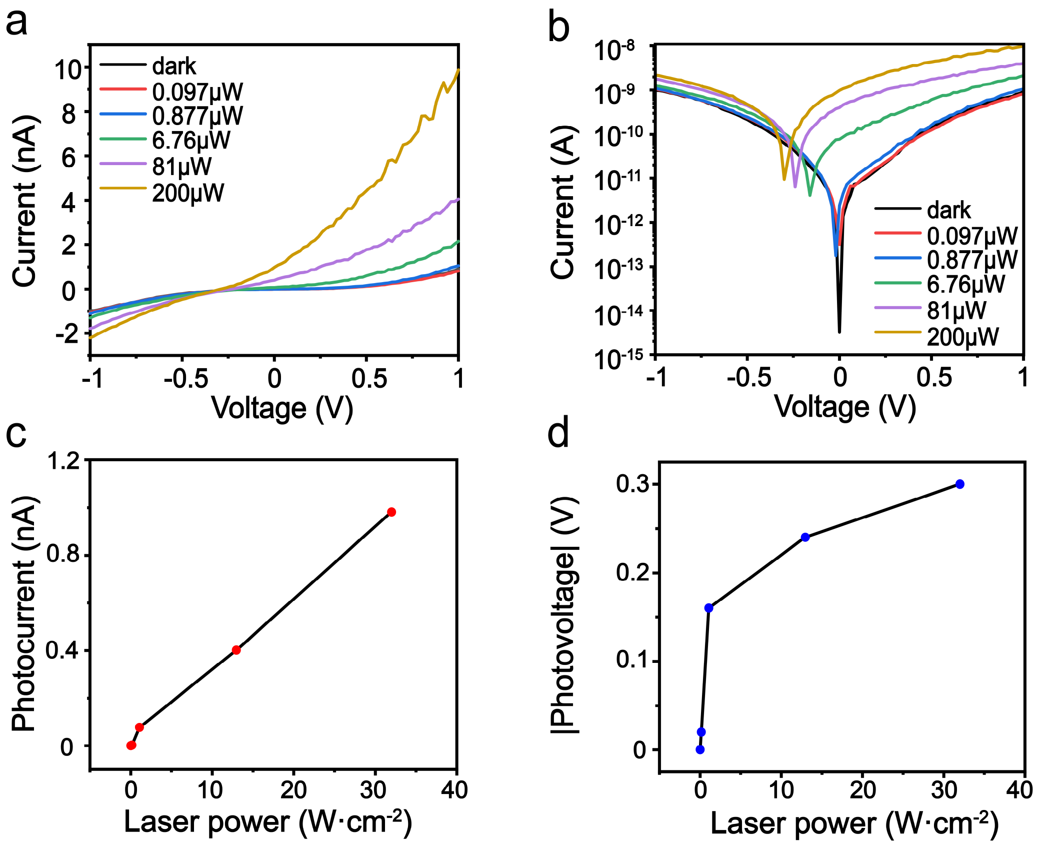

Supplementary Fig. 7 | Photovoltaic measurements with 488 nm laser. a, b, Linear (a) and log (b) scale *I-V* curves of the WS_2 nanotube. **c, d**, Photocurrent (c) and photovoltage (d) extracted from the *I-V* curves.

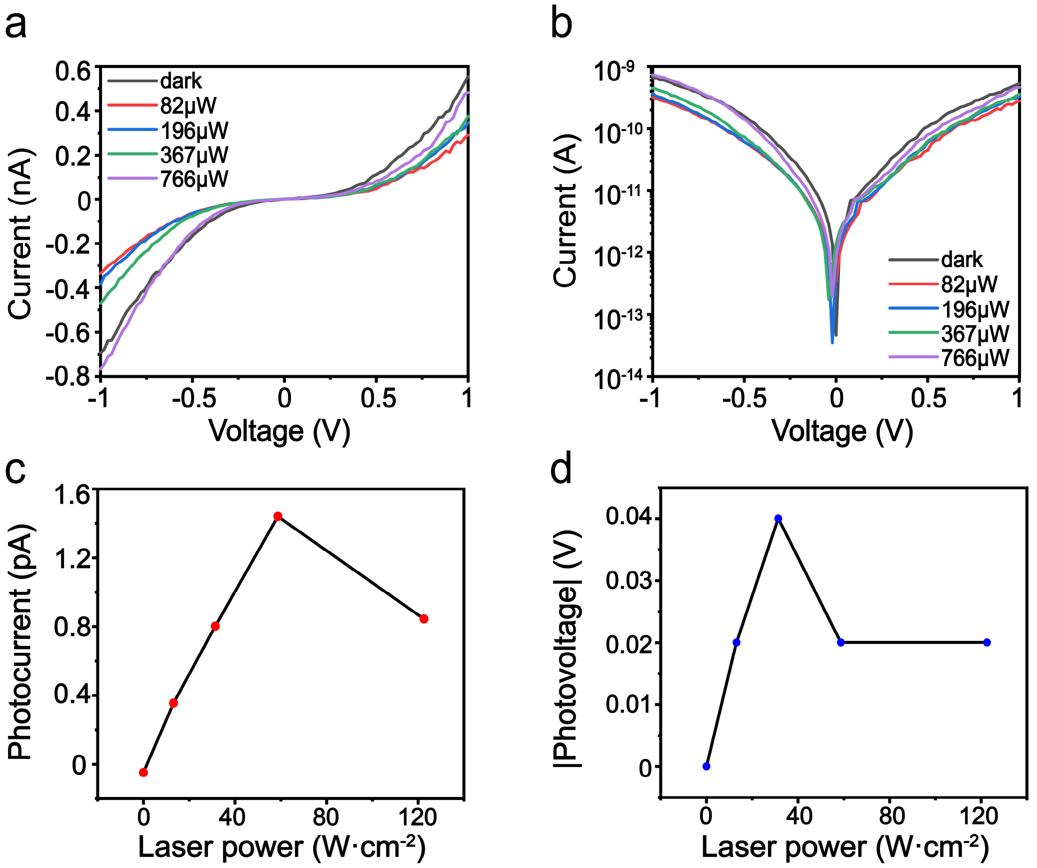

Supplementary Fig. 8 | Photovoltaic measurements with 325 nm laser. a, b, Linear (a) and log (b) scale *I-V* curves of the WS_2 nanotube. **c, d**, Photocurrent (c) and photovoltage (d) extracted from the *I-V* curves. Compared to measurement of visible light, the photoresponse has severely decreased probably due to UV induced device degeneration and carrier recombination.

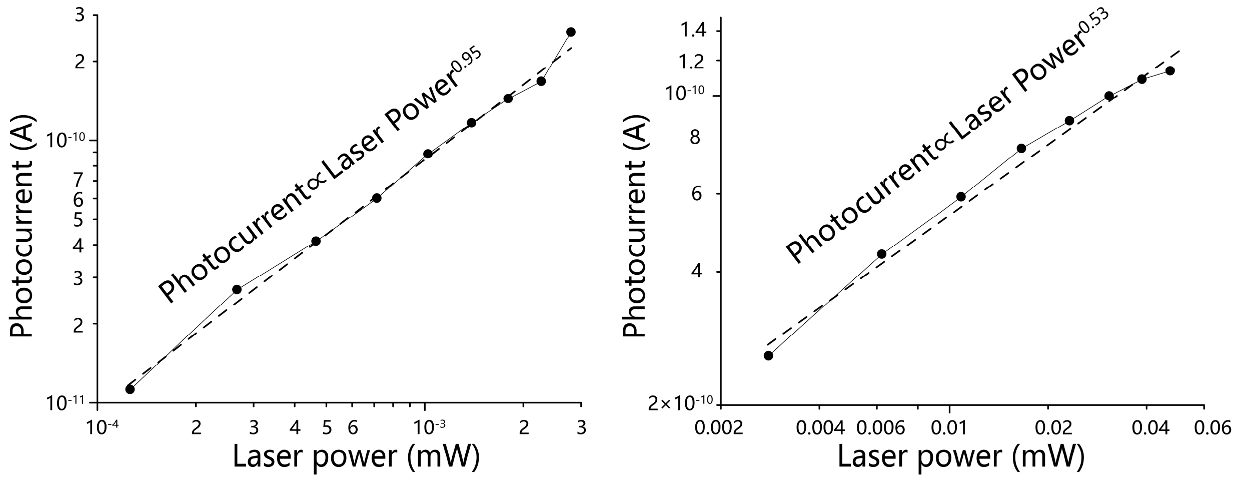

Supplementary Fig. 9 | Dependence of photocurrent on different laser power. The photocurrent shows good linearity with the small laser power (Photocurrent ∝ Laser Power$^{0.95}$) and degenerates to a near square-root dependence with larger laser power (Photocurrent ∝ Laser Power$^{0.53}$).

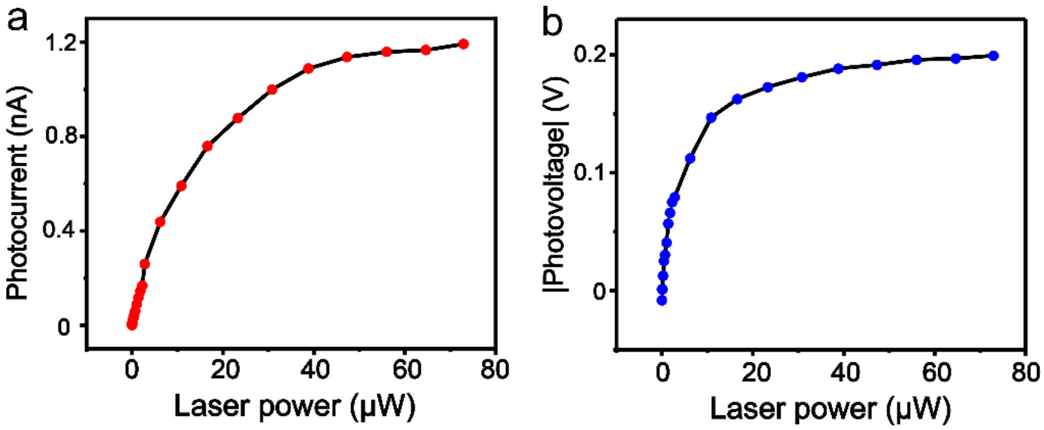

Supplementary Fig. 10 | Photocurrent and photovoltage with different laser power. a, Photocurrent with various laser power. **b**, Photovoltage with different laser power. Both the photocurrent and photovoltage reaches saturation with larger laser power as most photovoltaic devices do.

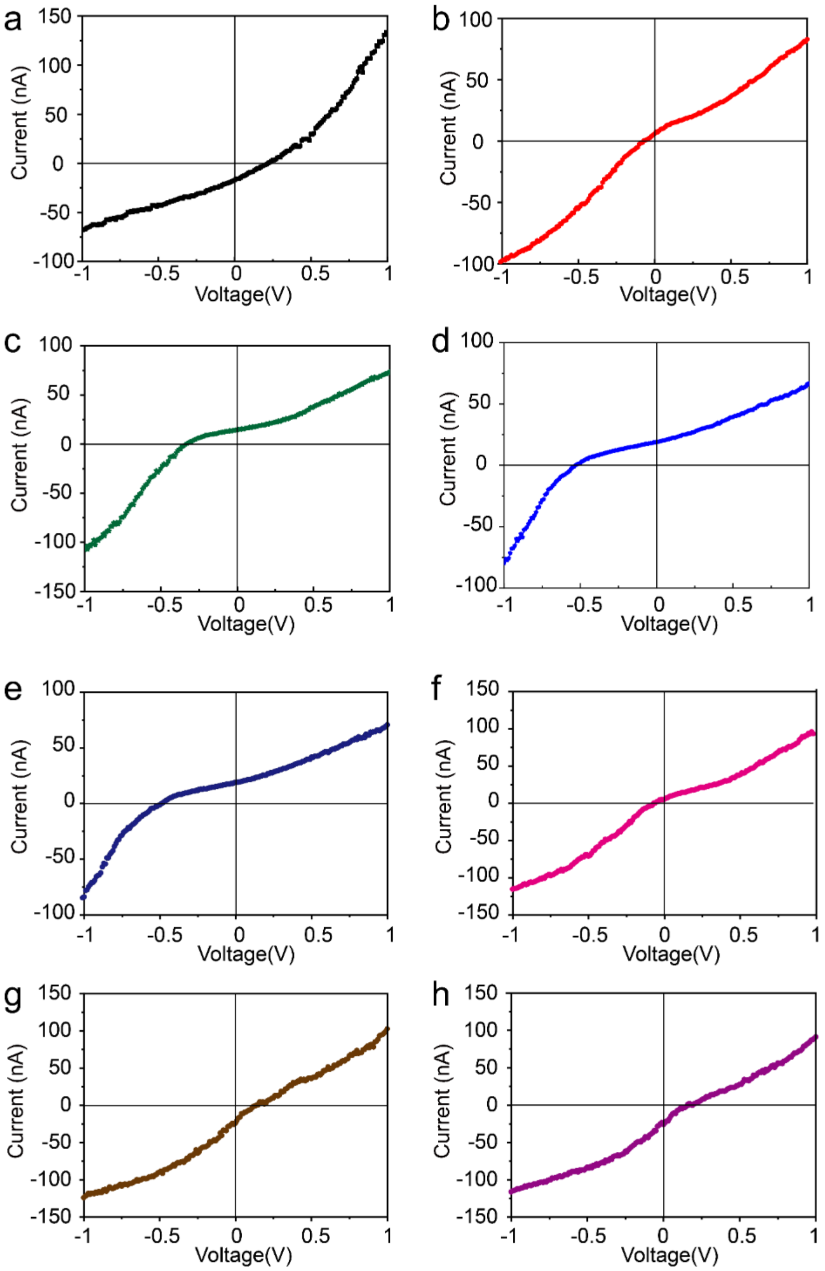

Supplementary Fig. 11 | *I-V* curves in linear scale. a, Initial *I-V* curve. **b-h**, *I-V* curves with a prior bias of -3 V (b), -4 V (c), -5 V (d, e), 3 V (f), 4 V (g), and 5 V (h).

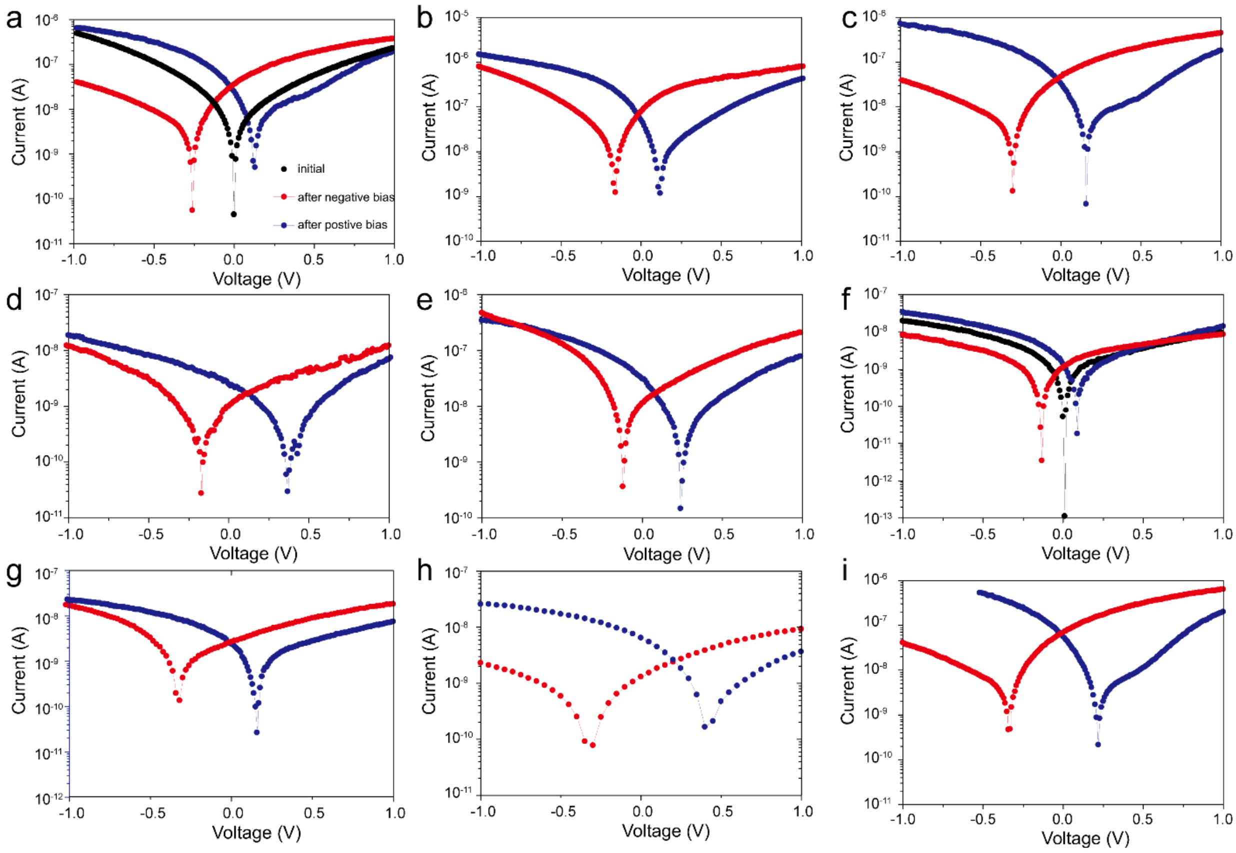

Supplementary Fig. 12 | Switchable photovoltaic effect in 9 WS_2 nanotube devices. For devices **a** and **f**, the initial photovoltaic effect is indistinctive (black), but was later enhanced by the negative (red) or positive (blue) bias. For the other devices (**b**, **c**, **d**, **e**, **g**, **h**, and **i**), the initial photovoltaic effect is distinct and is switchable by voltage bias.

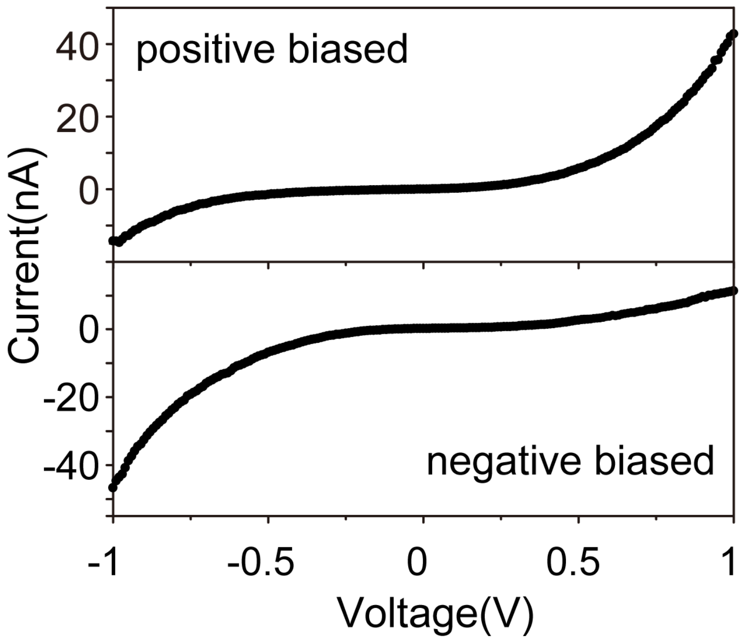

Supplementary Fig. 13 | *I-V* curves in dark after applying positive and negative bias. The IV curves show switchable rectification.

The determinative repeatable programmability of the photovoltaic effect is an important advance towards its practical application whether for energy transition or edge computing, otherwise the photovoltaic effect from multiple nanotubes with random and unpredictable direction would be neutralized. This work demonstrates that the photovoltaic effect is programmable, and its direction is determinative, therefore the overall photocurrent/voltage from the multiple nanotubes could be accumulated or enlarged using multiple nanotubes in parallel/series, as shown in Supplementary Fig. 14.

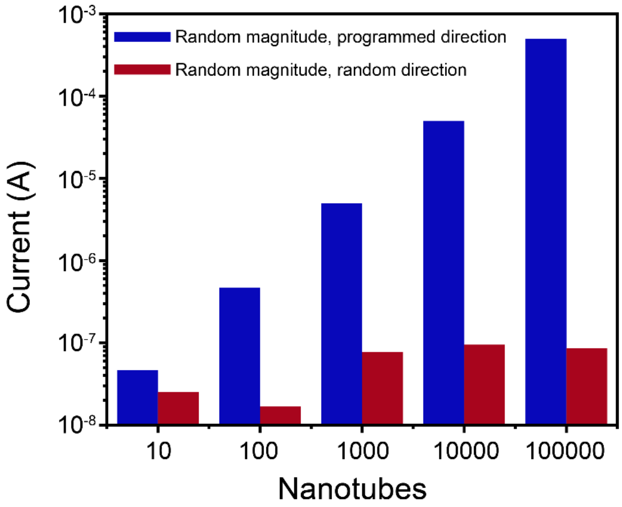

Supplementary Fig. 14 | Overall photocurrent of parallel photovoltaic devices with determinative/random direction. Compared to the random but fixed photovoltaic effect, the photovoltaic effect of WS_2 nanotube is programmable, and its direction is determinative, therefore the overall photocurrent from the multiple nanotubes could be accumulated.

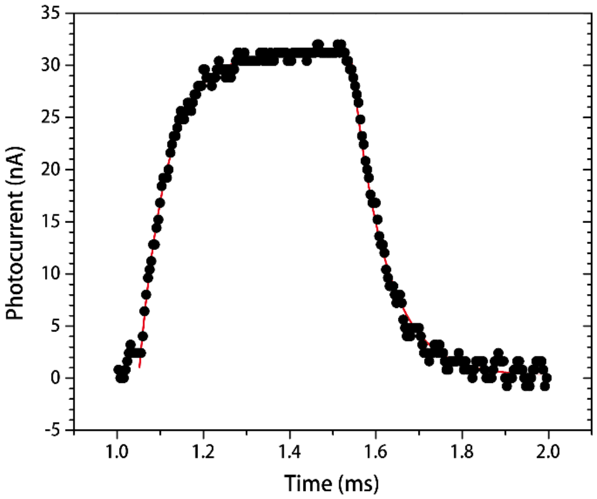

Supplementary Fig. 15 | Photo response to a single laser ON/OFF period. The rise time of 64 μs and the fall time of 77 μs can be extracted.

**Devices**	**Responsibility**	**Response time**	**Programmability**	**Range**	**Memory**	**Reference**
2D WS_2 (multilayer)	30 μA/(W/cm^2)	Raise: 7.8 ms<br>Fall: 37.2ms	No	Red to ultraviolet (bandgap 1.8 eV)	No	[Gao et.al., Adv. Electron. Mater. 7, 7, 2021]
2D MoS_2 (multilayer)	0.14 (A/cm^2)/(W/cm^2)	Not available	No	Red to ultraviolet (bandgap 1.4 eV)	No	[Wi et.al., ACS Nano, 8, 5, 2014]
1D carbon nanotube	0.22 nA/(W/cm^2)	Not available	No	infrared	No	[Yang et. al., Nat. Photon. 5, 11, 2011]
MoS_2/Si	3 mA/(W/cm^2)	Rise: 3 μs<br>Fall: 40μs	No	NIR to visible	No	[Wang et. al., Adv. Fun. Mater. 25, 19, 2015]
2D WSe_2	160 pA/(W/cm^2)<br>0.24 mA/W<br>0.2 mA/W	rise 12.3 ms<br>fall 11.4 ms	Yes	Red to ultraviolet (bandgap 1.54 eV)	No	[Groenendijk et. al., Nano Lett. 14, 10, 2014]<br>[Baugher et. al., Nat. Nanotechnol., 9, 4, 2014]<br>[Pospischil et. al. Nat. Nanotechnol., 9, 4, 2014]
$BiFeO_3$	8 pA/(W/cm^2)	Not available	Yes	Blue to ultraviolet (bandgap 2.74 eV)	Yes	[Guo et. al., Nat. Commun. 4, 1, 2013]
1D WS_2	0.2 (A/cm^2)/(W/cm^2)	Rise 64 μs<br>Fall 77 μs	Yes	Red to ultraviolet (bandgap 1.8 eV)	Yes	[Zhang et. al., Nature, 570, 7761, 2019]<br>This work

Supplementary Table. 1 | Photovoltaics of 2D MoS_2, 2D WS_2, 2D WSe_2, 1D WS_2, and $BiFeO_3$. Only photovoltaic performance is included.

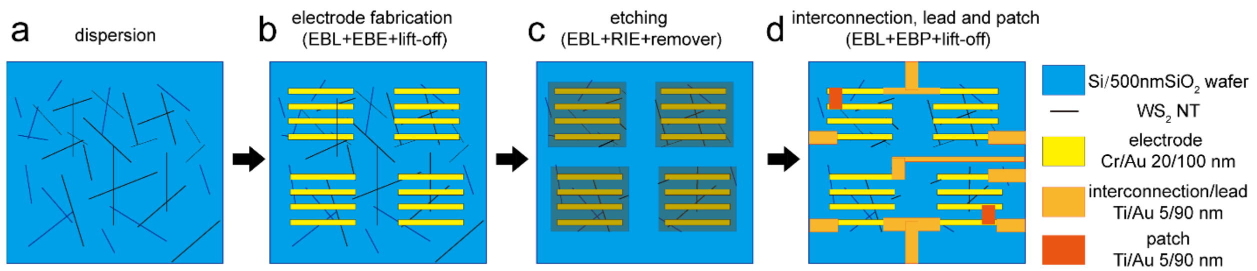

Supplementary Fig. 16 | Fabrication process of the WS_2 nanotube PV-RAM array. a, Dispersion of the WS_2 nanotubes. **b**, Fabrication of the electrodes by electron beam lithography, electron beam evaporation, and lift-off process. **c**, Etching of WS_2 nanotube out of the PV-RAM area. **d**, Interconnection of the PV-RAM array, leads to the pad, and patches of the PV-RAM.

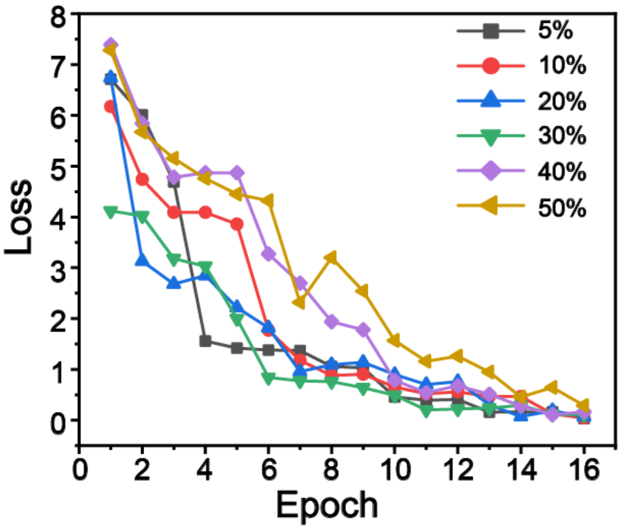

Supplementary Fig. 17 | Learning curve of loss with different levels of noise. The loss decreases with the training process.

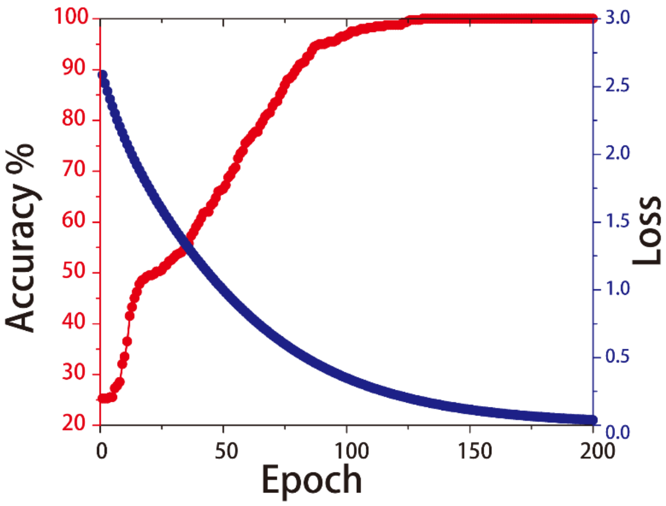

Supplementary Fig. 18 | The training process of the neural network with continuous weight. Red: accuracy. Blue: Loss function. The noise level is set to 0. The loss decreases and the accuracy increases with the training process.

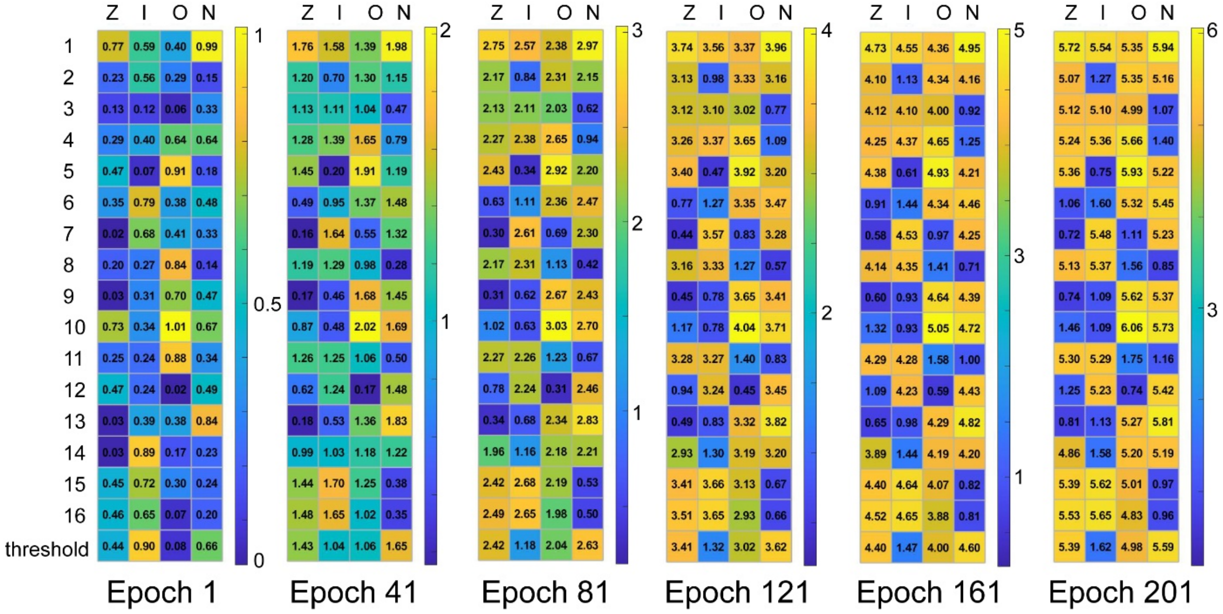

Supplementary Fig. 19 | The trained weights of the artificial neural network. The weights are adjusted using the decreasing gradient algorithm.

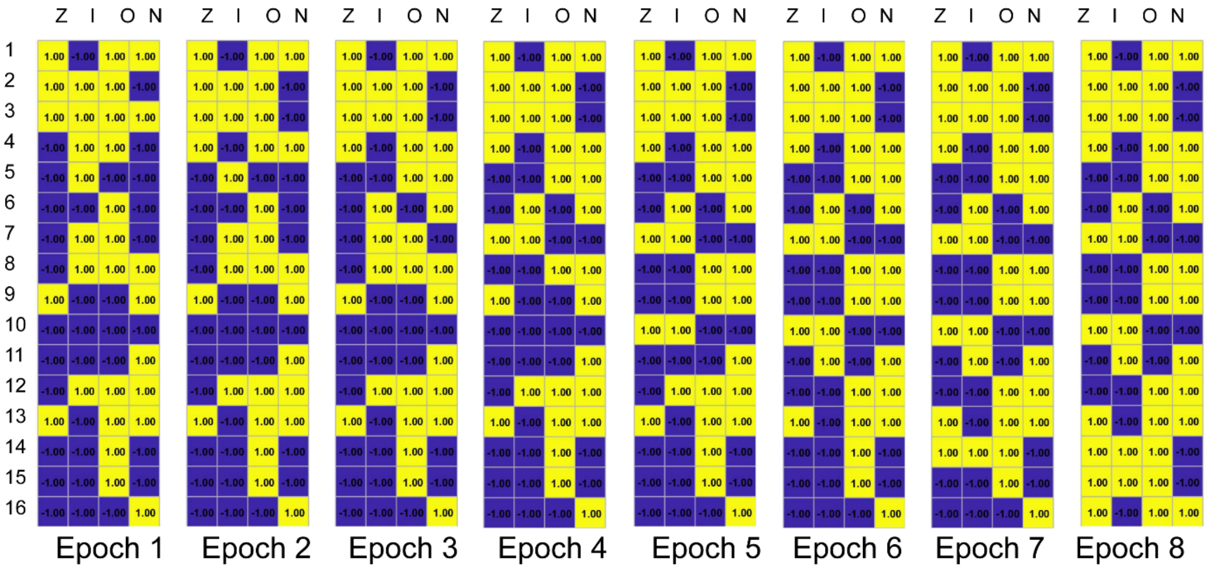

Supplementary Fig. 20 | The trained weights of the binary artificial neural network. The weights are binary and have discrete distributions of 1 and -1.

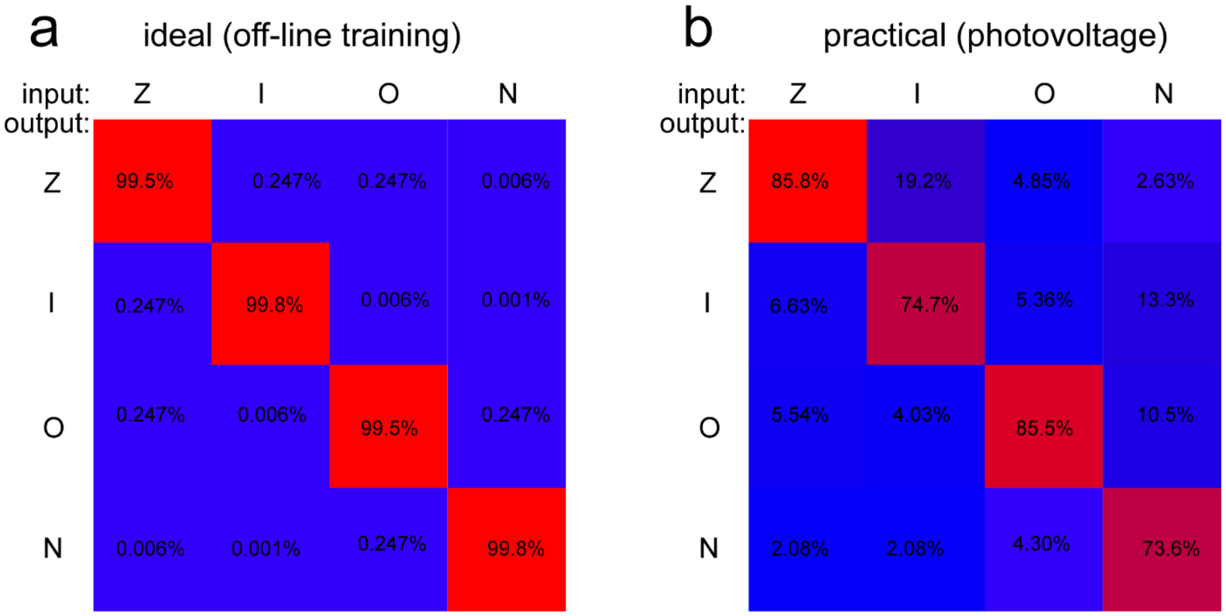

Supplementary Fig. 21 | Classification of the images. a, Calculated probabilities from the one-hot encoding through the offline training. **b**, Probabilities from the one-hot encoding of the measured photovoltage.

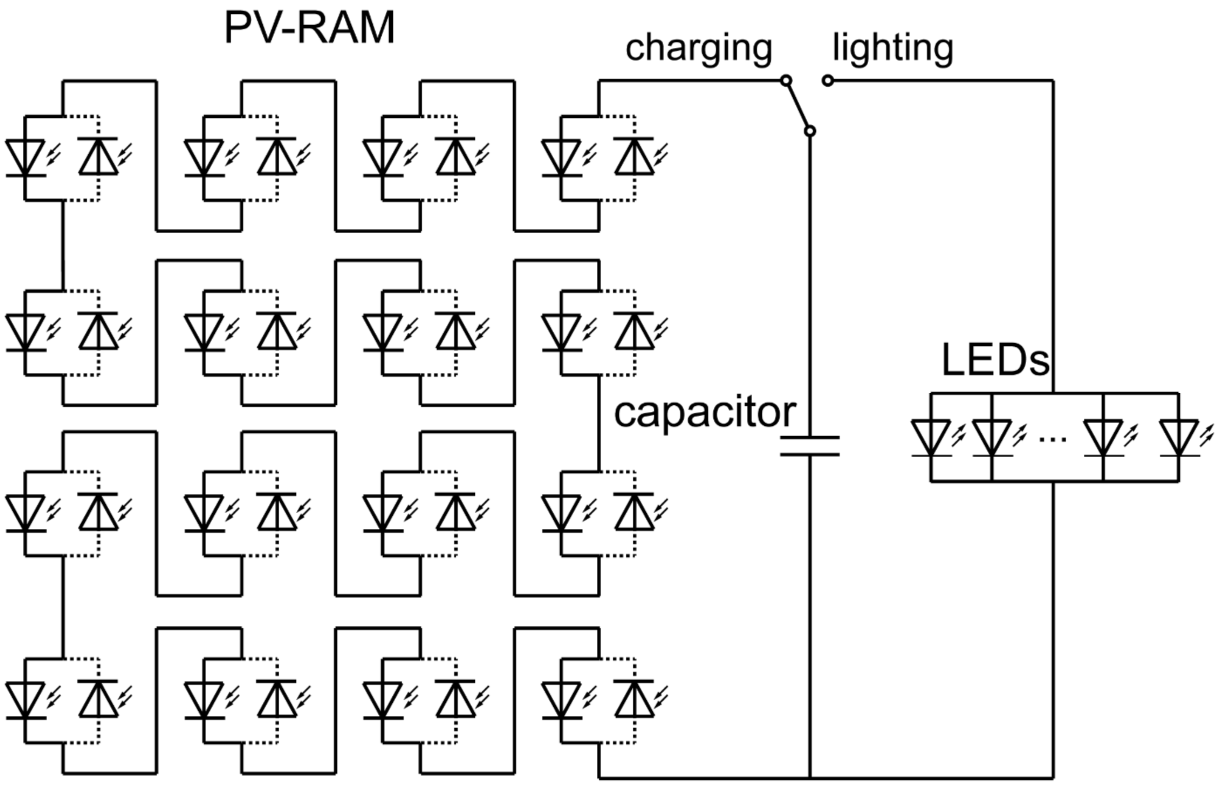

Supplementary Fig. 22 | Equivalent circuits of the PV-RAM array. The array can charge the capacitor and drive LED patterns.

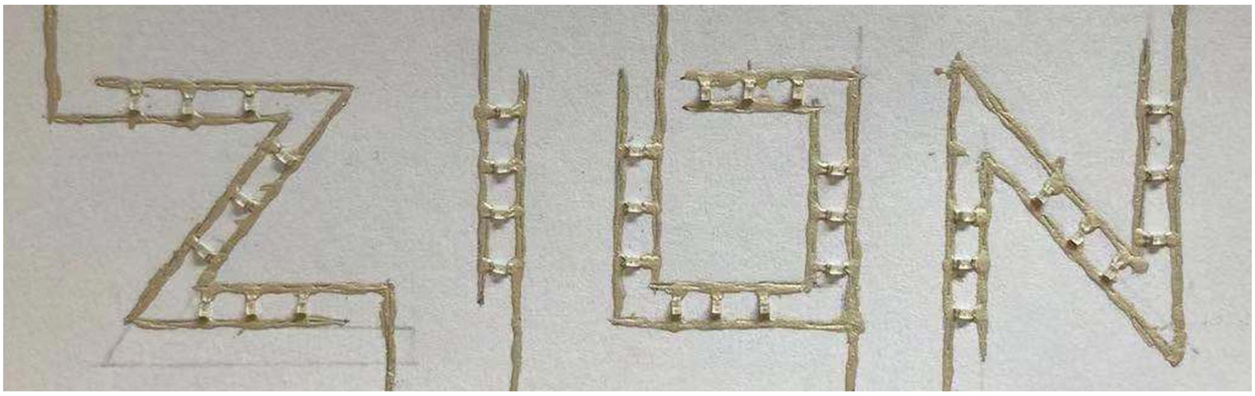

Supplementary Fig. 23 | LED patterns of ‘Z’, ‘I’, ‘O’, and ‘N’. The LEDs are fixed by electrargol on office paper as the flexible substrate.

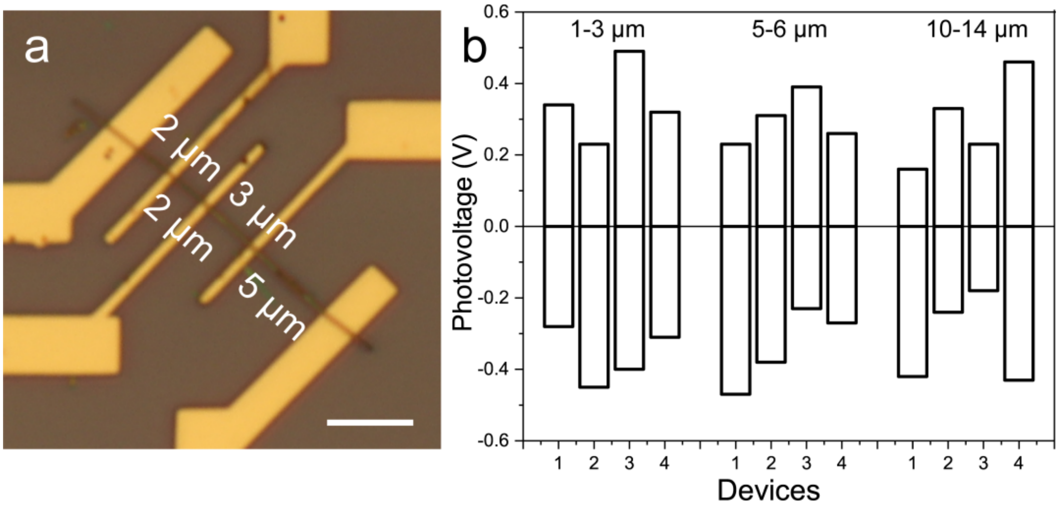

Supplementary Fig. 24| Photovoltaic effect of WS_2 nanotube devices with different channel lengths. **a**, Optical image of WS_2 nanotube devices. Scale bar: 5 µm. **b**, Photovoltage of WS_2 nanotube with different wavelength. The diameter of the laser spot is set about 15 µm to cover the whole devices.

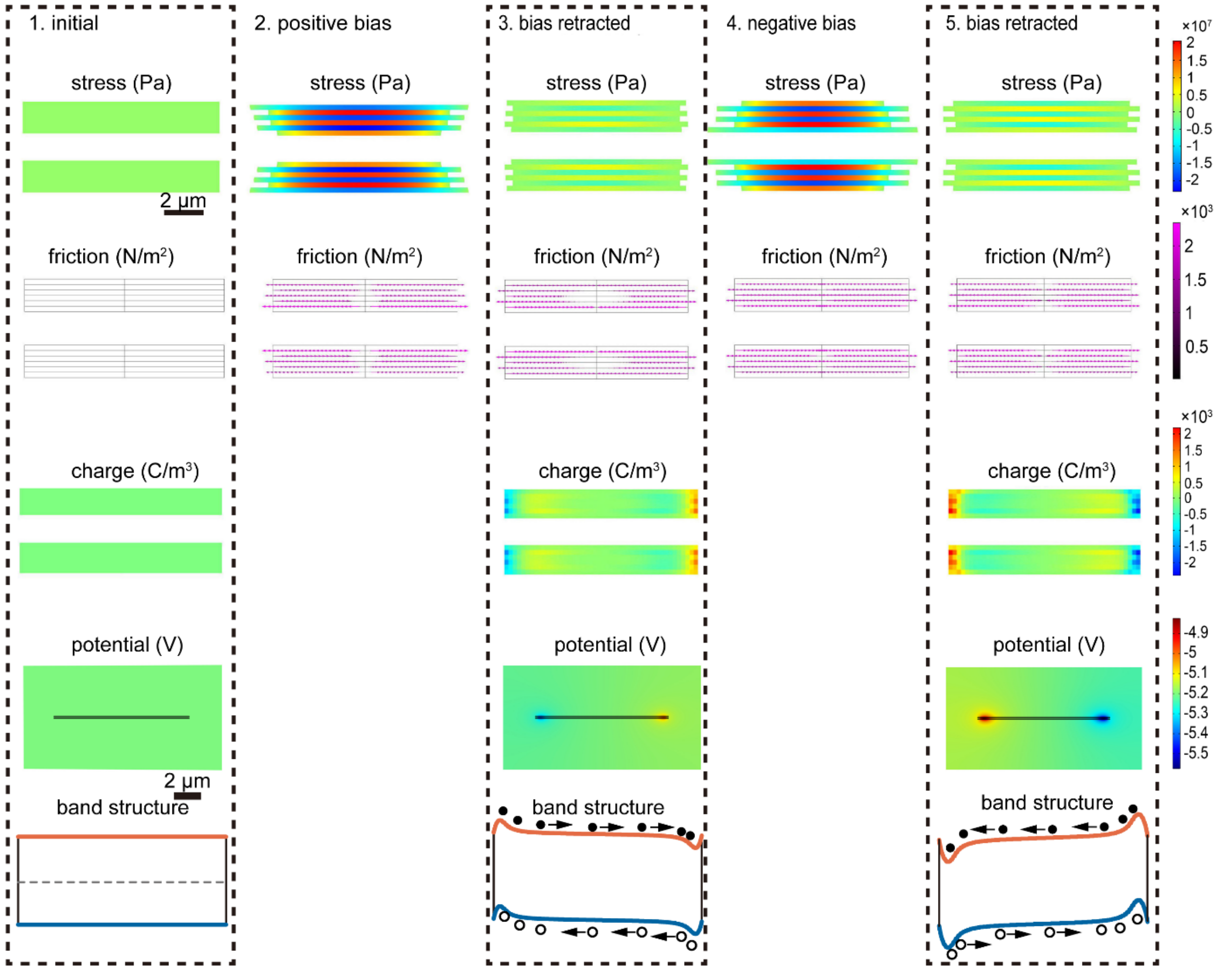

Supplementary Fig. 25| The simulated sliding process between WS_2 layers that generates ferroelectricity. Stress/deformation, friction force (normalized by area), charge distribution, voltage potential, and band structure with indications of the movement of the photogenerated carriers (solid: electrons, hollow: holes) in deformed WS_2 nanotube. For visualization of stress, friction, and charge, the thickness of the layer is magnified from 0.7 nm to 175 nm (250 times). The deformation in the plot of stress is magnified 2000 times.

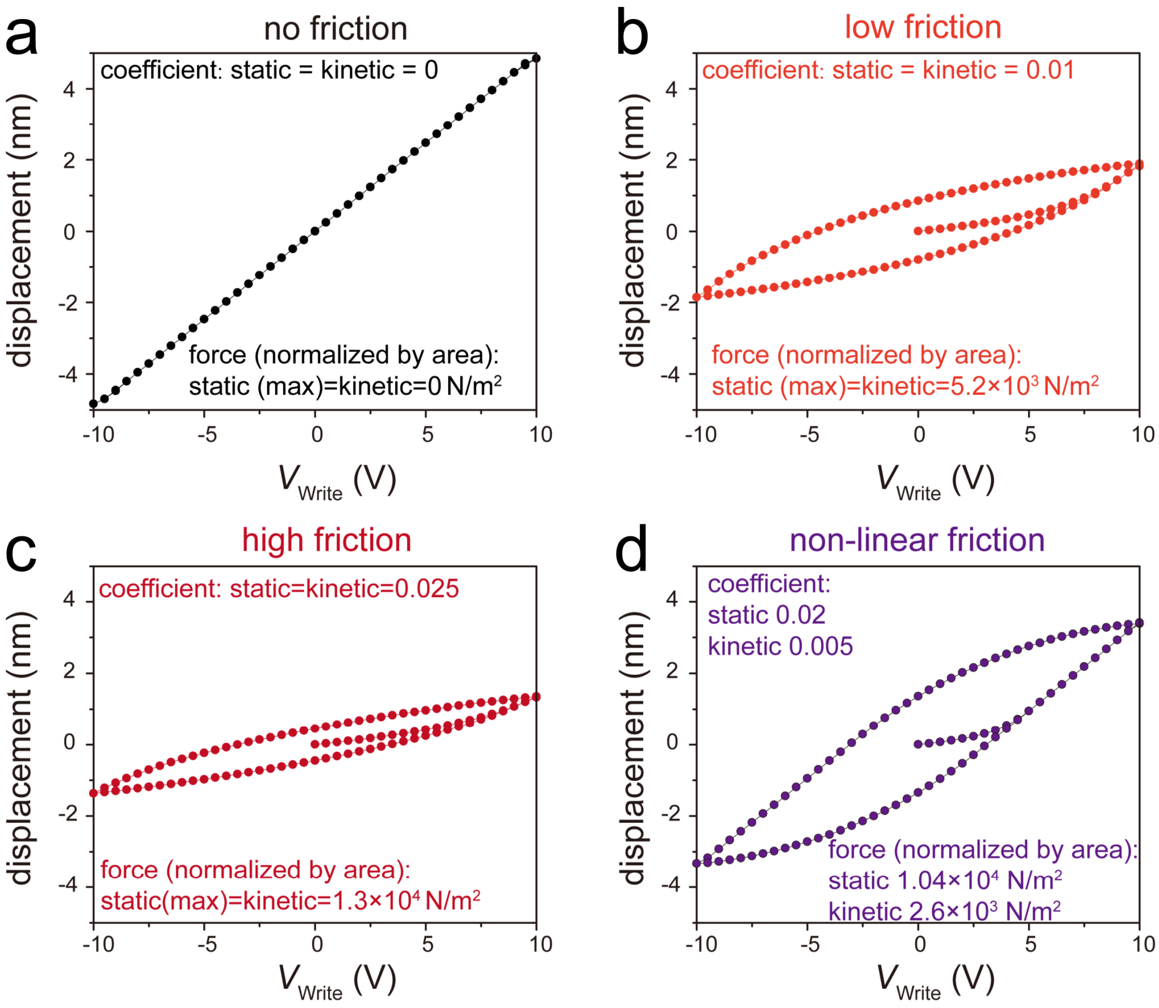

Supplementary Fig. 26| Model calculations of interlayer sliding under different friction conditions. **a**, No friction. **b**, Low friction (friction coefficient of 0.01). **c**, High friction (friction coefficient of 0.025). **d**, Non-linear friction: the static and kinetic friction coefficients were set differently (static friction coefficient of 0.02, kinetic friction coefficient of 0.005). A radial pressure was set 5.2×10^5 Pa.

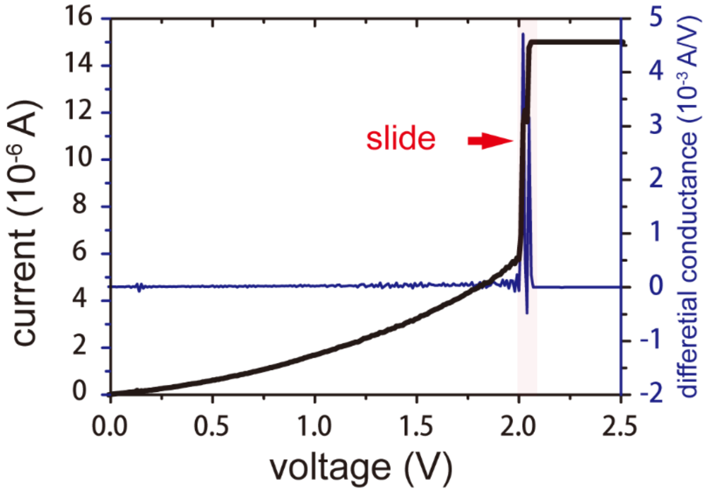

Supplementary Fig. 27| ***I-V*** **curve and differential conductance of the** $\mathbf{WS_2}$ **nanotube.** Conductivity is stable at a small voltage and shows an abrupt change at a large voltage of about 2.1 V.

To further confirm the spontaneous electrical polarization, we conduct the KPFM on the WS_2 nanotube device as fabricated and after applying voltage bias. As is shown in Supplementary Fig. 27, despite the fluctuations of the measured potential along the middle of the channel, our results show that the voltage potential distribution is changed according to the applied bias, showing opposite trends of the slope with 5V and -5V. It should be noted, however, that due to the long-range detection scheme of KPFM, the electrostatic interaction from the device includes both contributions of the nanotube and the contacts to the tip and the cantilever, which limits the spatial and potential resolution.

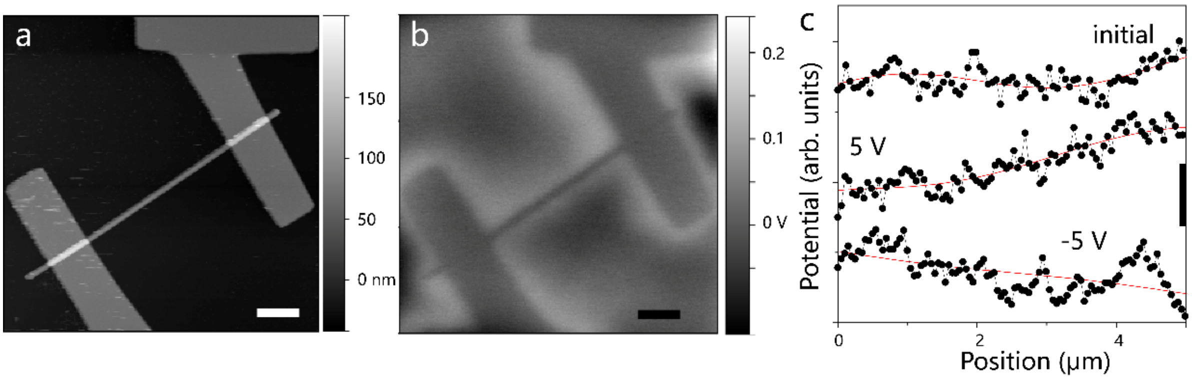

Supplementary Fig. 28| Spontaneous electrical polarization of WS_2 nanotubes under KPFM. a, Atomic force microscopy image of the WS_2 nanotube device. Scale bar: 2 μm. **b**, Potentials of the WS_2 nanotube device. Scale bar: 2 μm. **c**, Potentials of the as fabricated WS_2 nanotube device, and that after applying positive/negative bias. Scale bar: 25 mV.

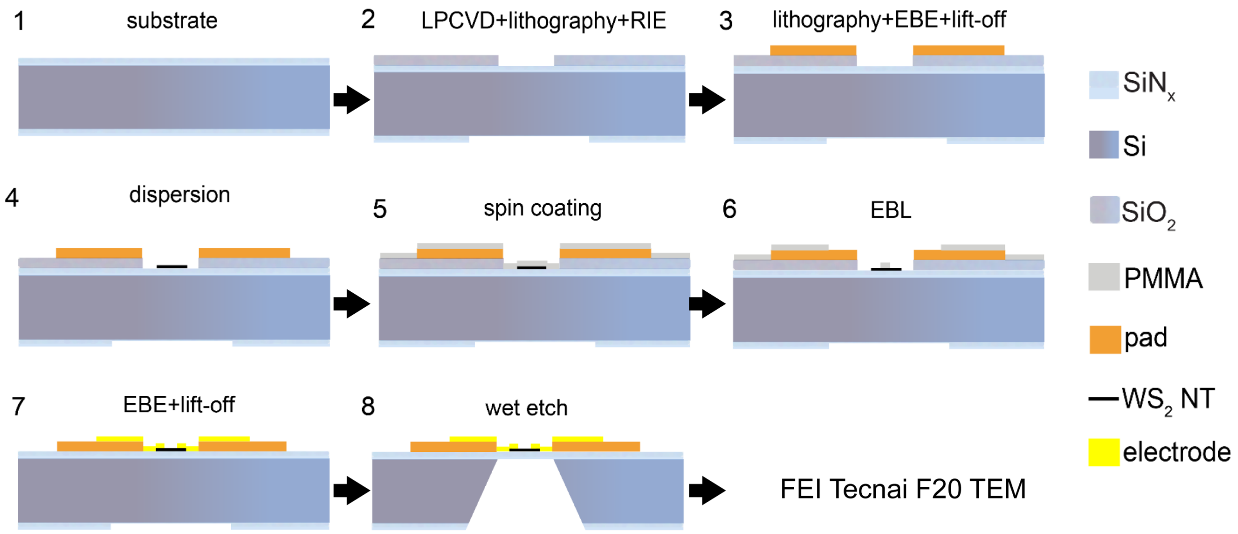

Supplementary Fig. 29| Fabrication process of the WS_2 nanotube for the in-situ TEM characterization. The process includes low pressure chemical vapor deposition (LPCVD) + lithography + reactive ion etching (RIE), lithography + electron beam evaporation (EBE) + lift-off, dispersion, spin coating, EBL, EBE + lift-off, wet etch, followed by the in-situ TEM characterization.

Supplementary Text

Given the more intense photoresponse at the ends of the WS_2 nanotube, our initial speculation is that the photoresponse should be originated from the Cr-WS_2 or Ti-WS_2 Schottky barriers, rather than the van der Waals sliding ferroelectricity. However, the Schottky barrier should generate the opposite photovoltaic effect at the two ends in fixed directions, UNLESS the polarization inside the WS_2 nanotube is reversed. In fact, for the simulative potential distribution results of Supplementary Fig. 24, the boundary condition of Schottky contact was added, the contact fixes the voltage potential at the surface and the voltage potential show back-to-back slopes at the ends. Such back-to-back potential slopes were reported in the previous study[51], which reveals the monopolization of the photovoltaic effect at the thickness modulated MoS_2. The back-to-back potential slopes can generate a homogeneous photovoltaic effect only in the preponderant direction.
