# Supplementary material for: Mesoscopic sliding ferroelectricity enabled photovoltaic random access memory for material-level artificial vision system
Source: Nat Commun. 2022 Sep 14;13:5391. doi: 10.1038/s41467-022-33118-x (PMC9474805; doi:10.1038/s41467-022-33118-x)
Supplement: Supplementary file 2 — Description of Additional Supplementary Files [file 41467_2022_33118_MOESM2_ESM.pdf]

Description of Additional Supplementary Files

File Name: Supplementary Movie 1
Description: The simulated sliding process between WS_2 layers with applied bias (stress and deformation). Note that for visualization, the thickness of the layer is magnified from 0.7 nm to 175 nm, and the deformation is magnified by 2000 times.

File Name: Supplementary Movie 2
Description: The simulated friction during the sliding process between WS_2 layers.
